# Supplementary material for: Formulation Studies with Cyclodextrins for Novel Selenium NSAID Derivatives
Source: Int J Mol Sci. 2024 Jan 26;25(3):1532. doi: 10.3390/ijms25031532 (PMC10855879; doi:10.3390/ijms25031532)
Supplement: Supplementary file 1 [file ijms-25-01532-s001.zip › ijms-2798001-supplementary.pdf]

Table of Contents

Characterization – NMR spectra<br>Compound, compound + dimethyl sulfone, compound + β-CD, compound + γ-CDs		
**Series I – Selenoesters**		
I.3e	....	Figures S1-S4
I.4a	....	Figures S5-S8
I.4b	....	Figures S9-S12
I.4d	....	Figures S13-S16
I.4e	....	Figures S17-S20
**Series II – Diacyl Diselenides**		
II.1	....	Figures S21-S24
II.2	....	Figures S25-S28
II.3	....	Figures S29-S32
II.4	....	Figures S33-S36
II.5	....	Figures S37-S40
**Complex II.5:β-CD**		
^{1}H-NMR spectra of molar fractions	....	Figure S41
**Computational data**		
Docking score data	....	Tables S1-S2
Energy of the selected compounds	....	Table S3

ଓ Characterization – NMR spectra

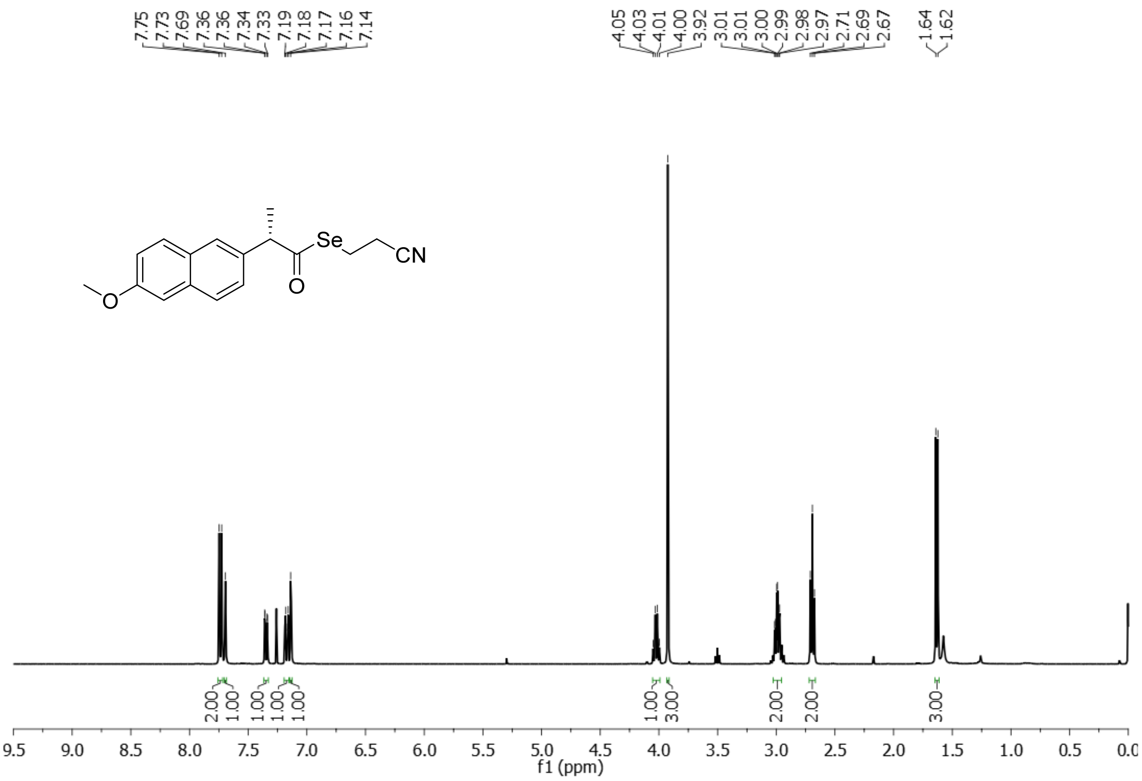

Figure S1. ^{1}H-NMR spectrum of compound **I.3e**.

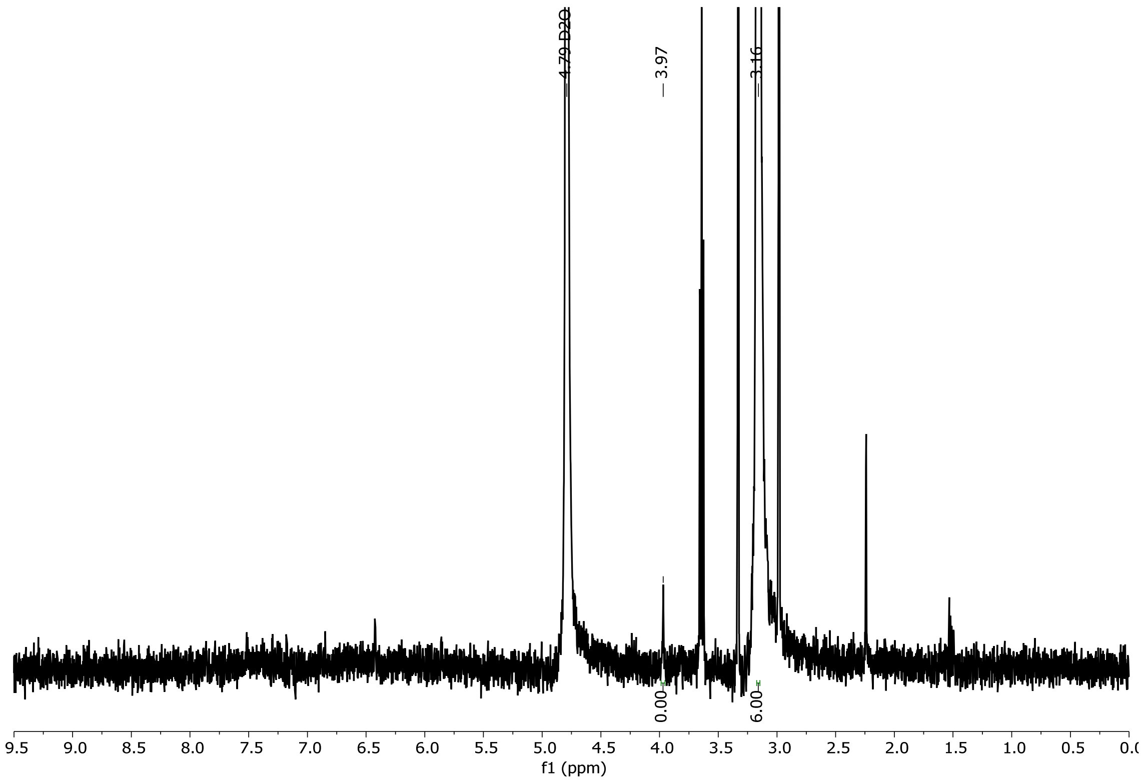

Figure S2. ^{1}H-NMR spectrum of compound **I.3e** and dimethyl sulfone.

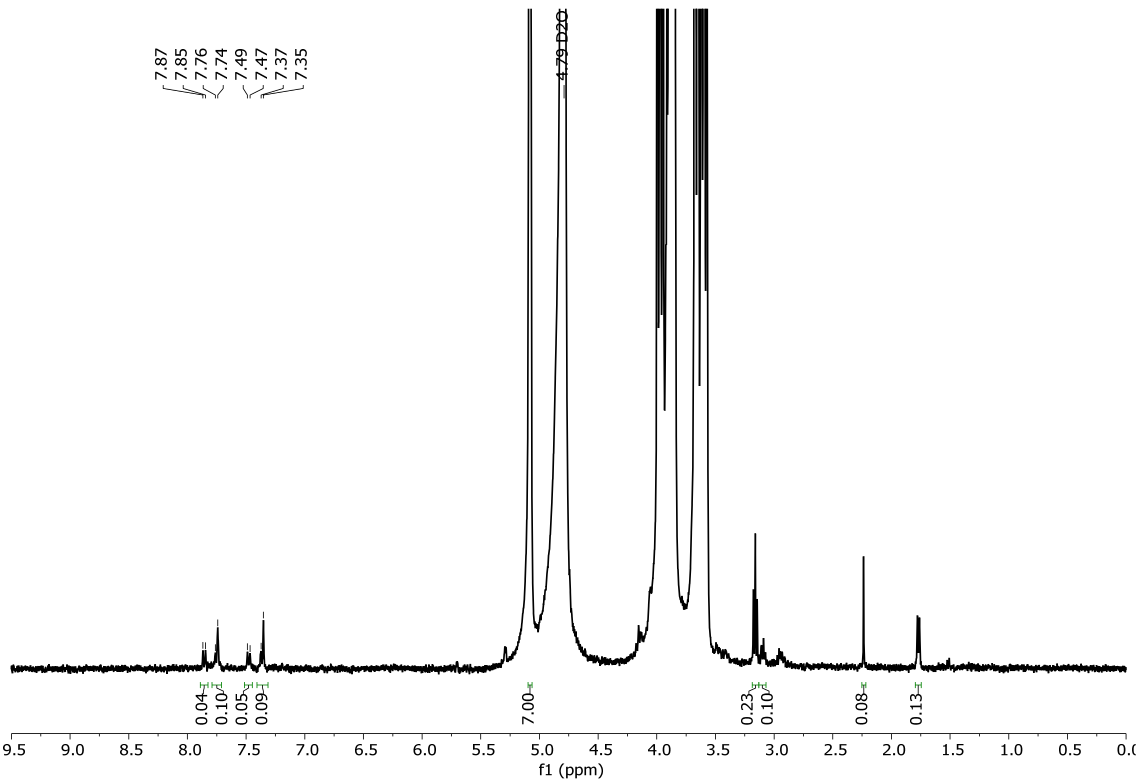

Figure S3. ^{1}H-NMR spectrum of compound **I.3e** and β-CD.

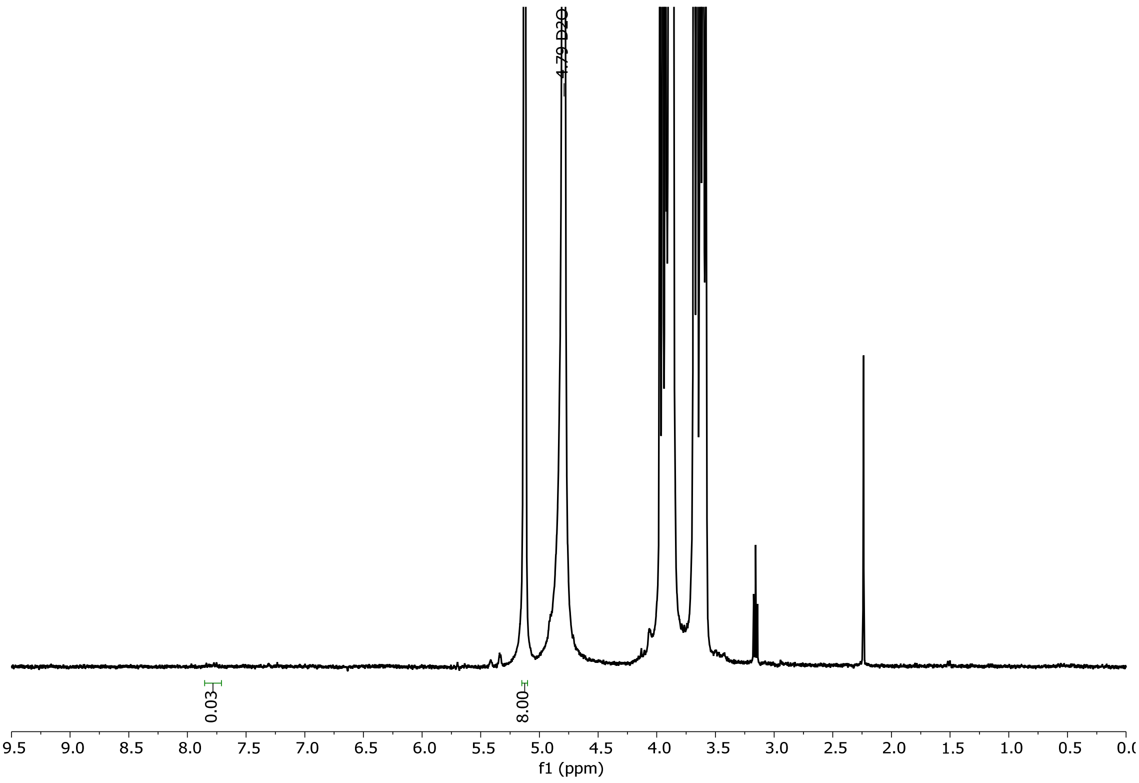

Figure S4. ^{1}H-NMR spectrum of compound **I.3e** and γ-CD.

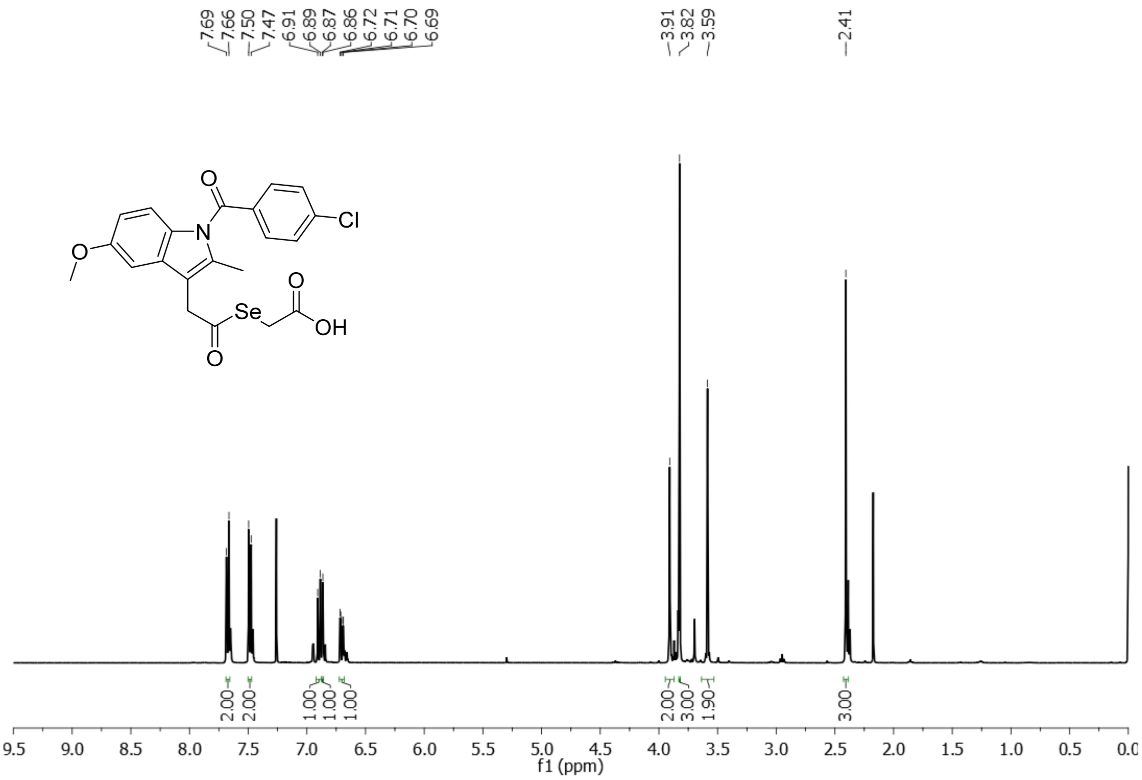

Figure S5. ^{1}H-NMR spectrum of compound **I.4a**.

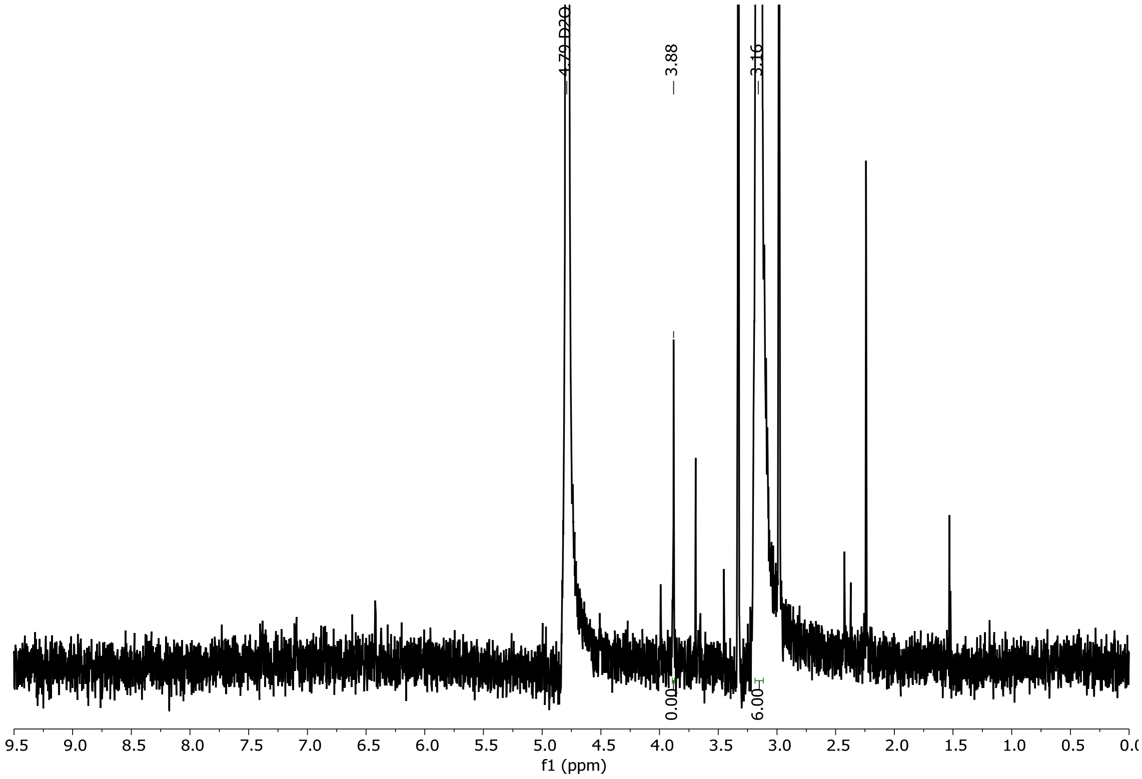

Figure S6. ^{1}H-NMR spectrum of compound **I.4a** and dimethyl sulfone.

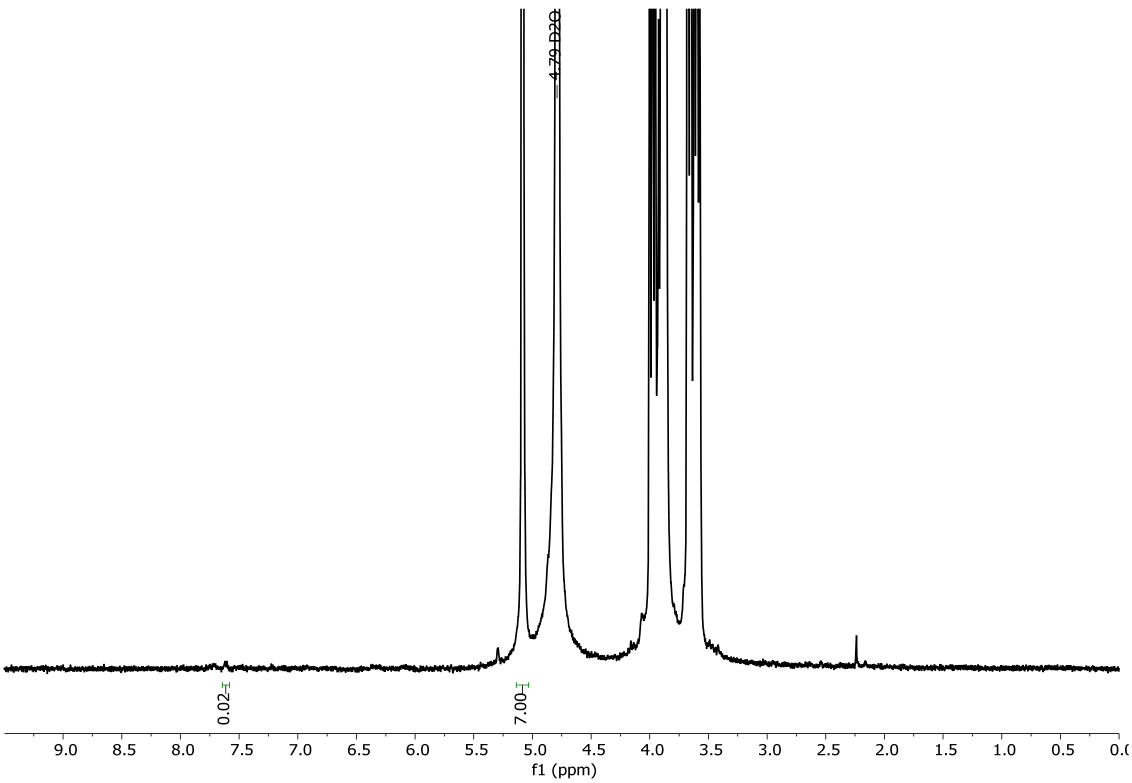

Figure S7. ^{1}H-NMR spectrum of compound **I.4a** and β-CD.

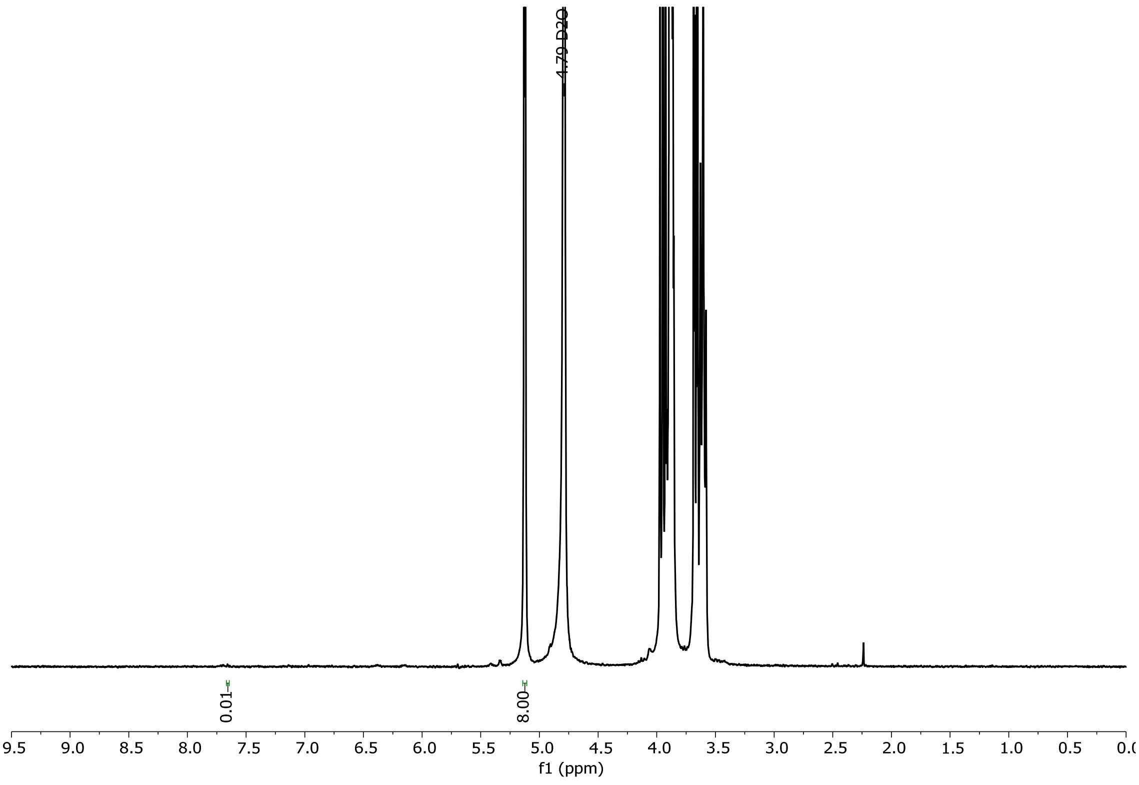

Figure S8. ^{1}H-NMR spectrum of compound **I.4a** and γ-CD.

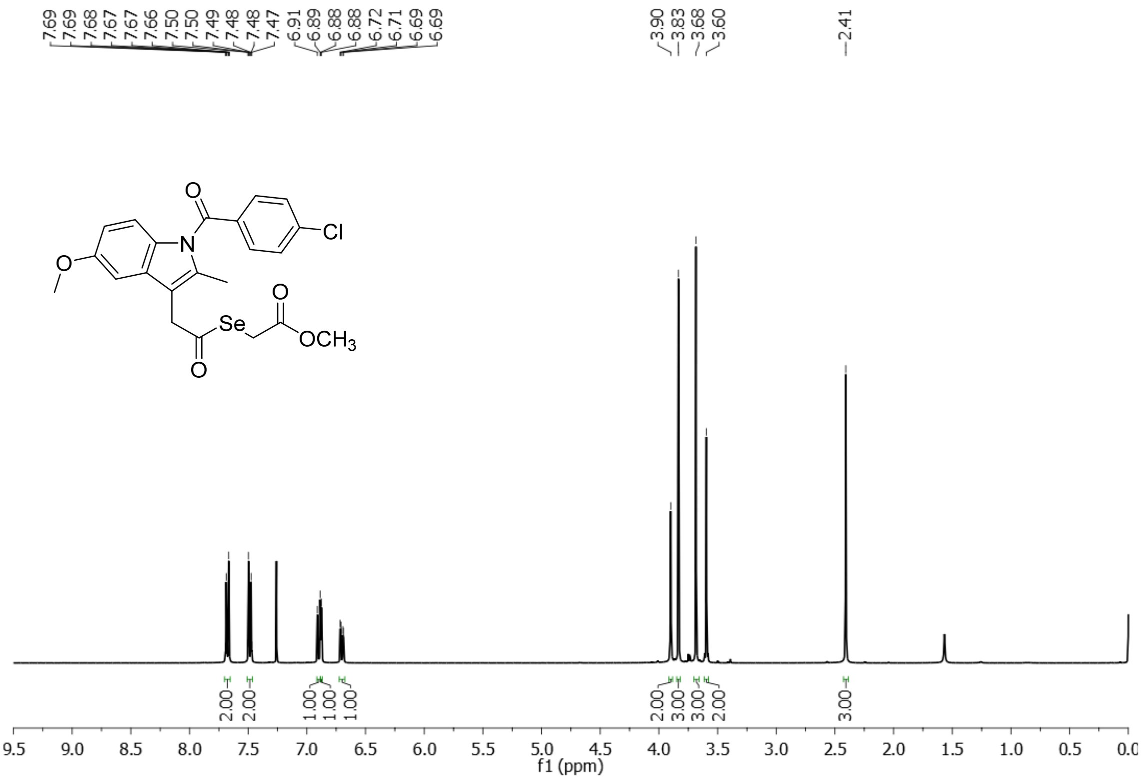

Figure S9. ^{1}H-NMR spectrum of compound **I.4b**.

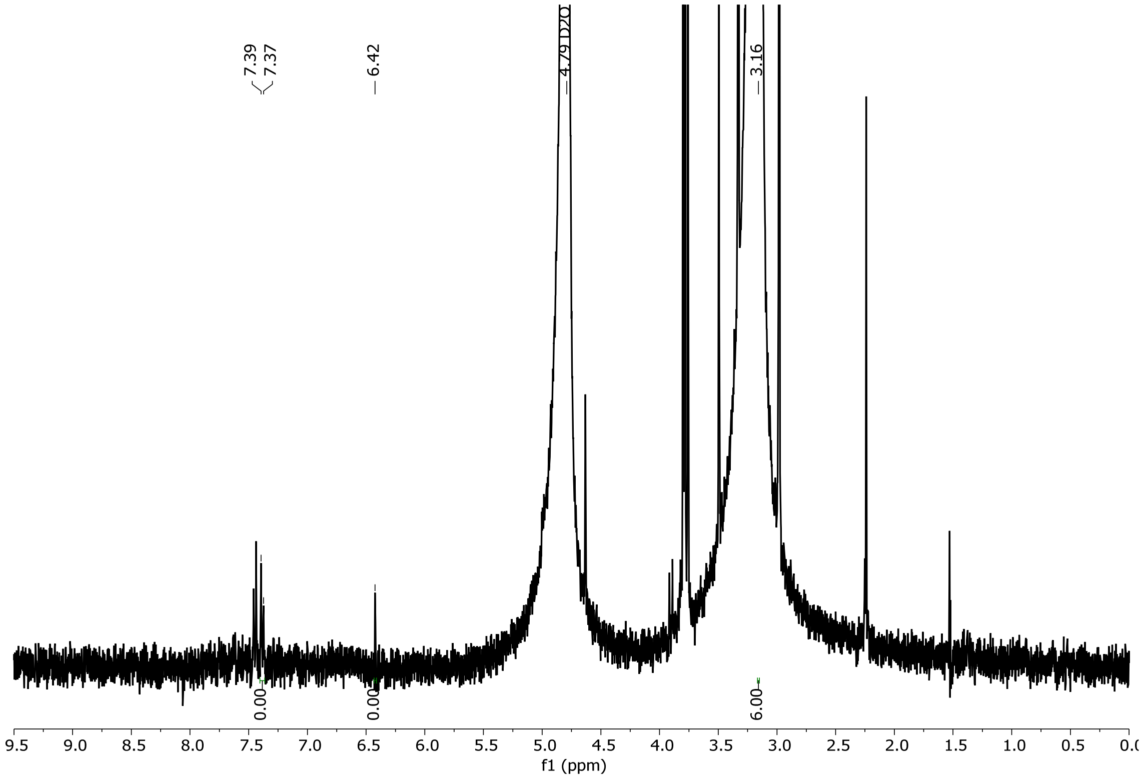

Figure S10. ^{1}H-NMR spectrum of compound **I.4b** and dimethyl sulfone.

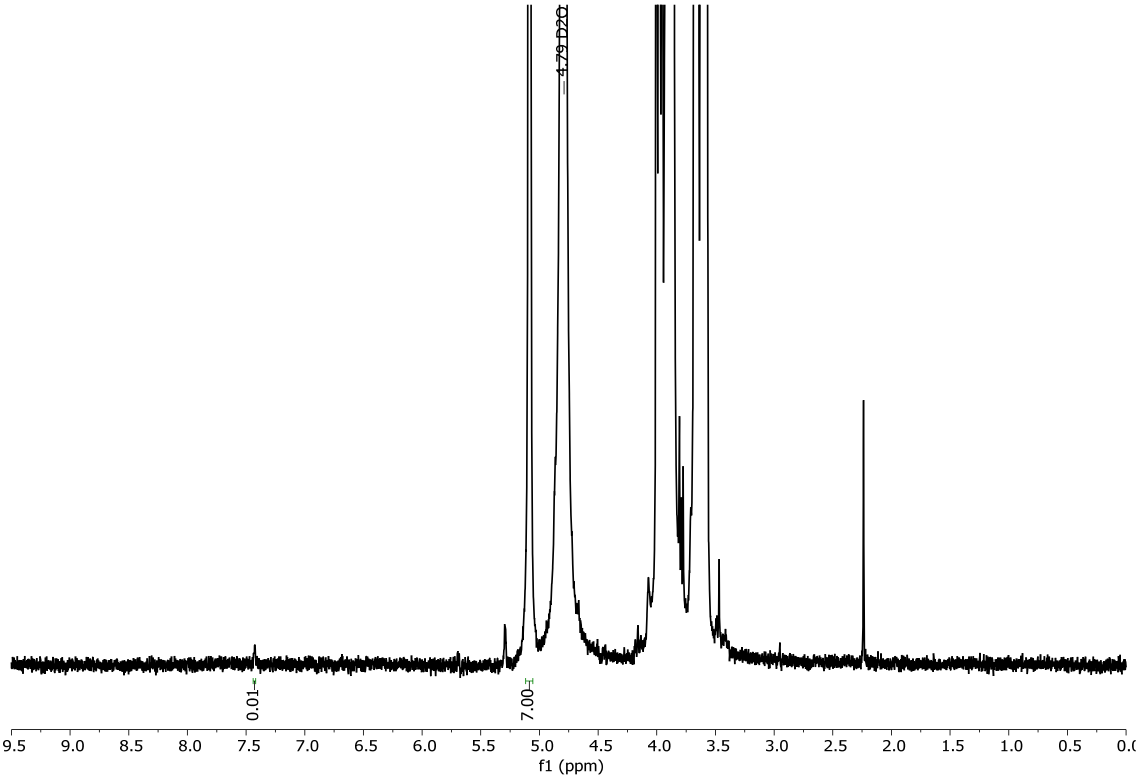

Figure S11. ^{1}H-NMR spectrum of compound **I.4b** and β-CD.

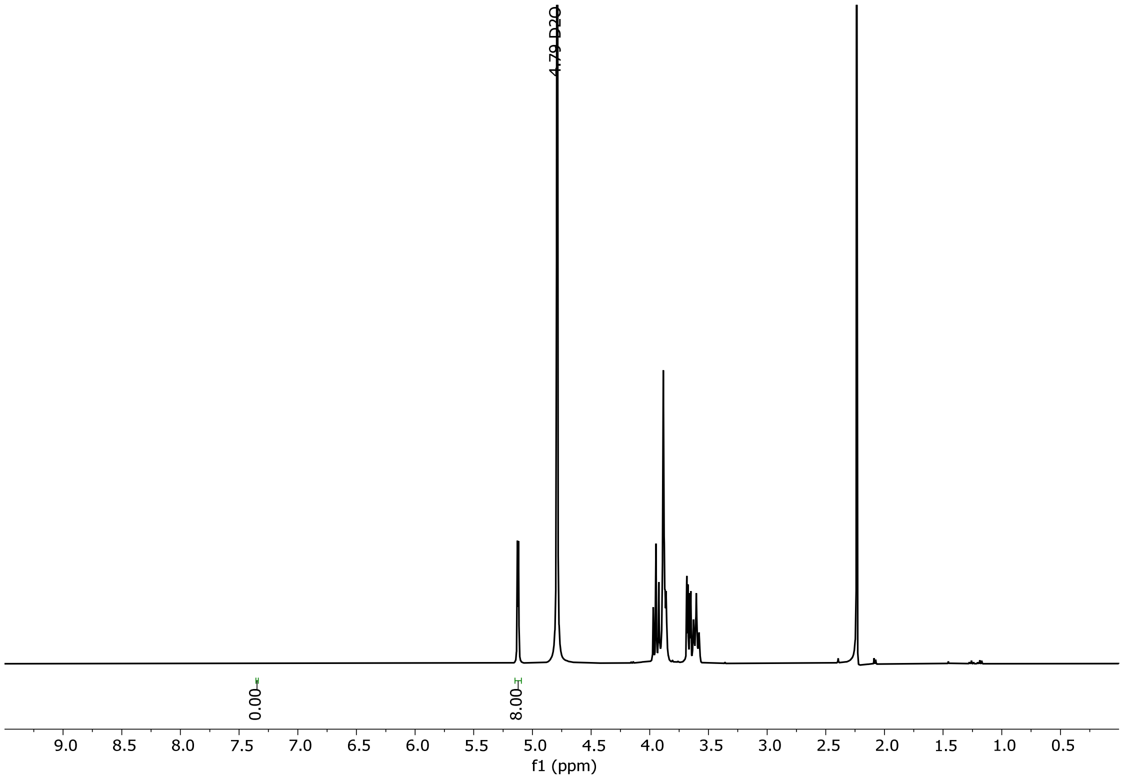

Figure S12. ^{1}H-NMR spectrum of compound **I.4b** and γ-CD.

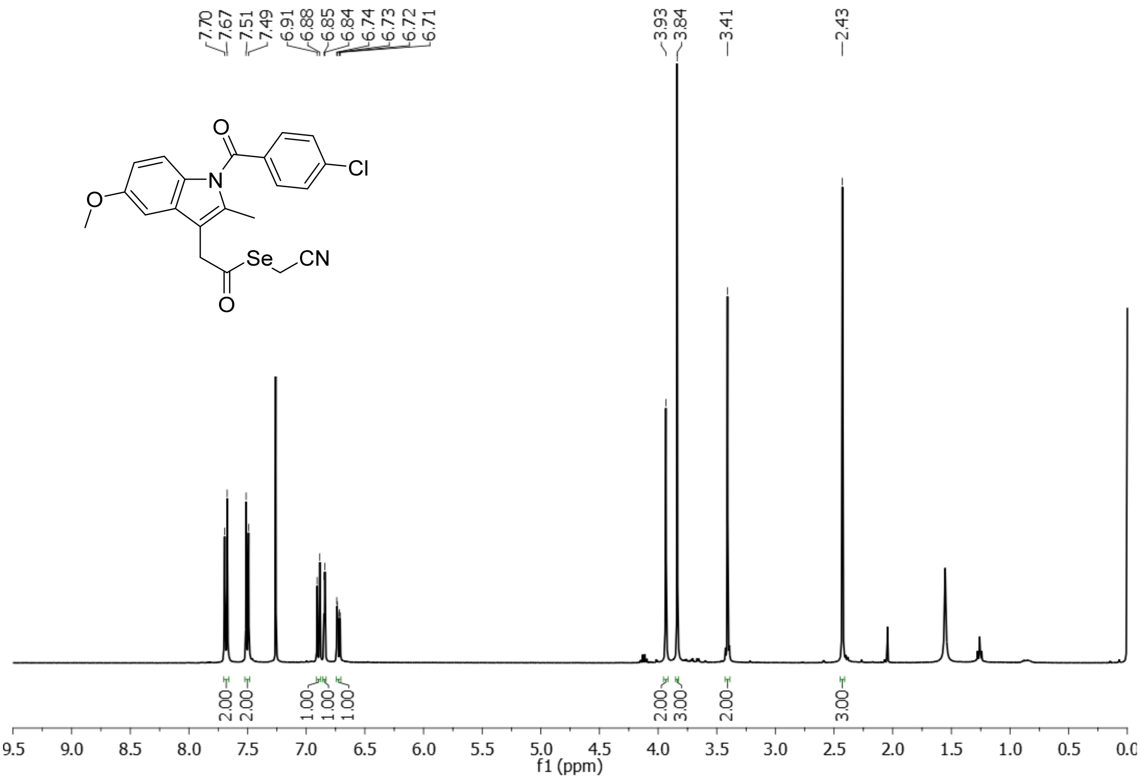

Figure S13. ^{1}H-NMR spectrum of compound **I.4d**.

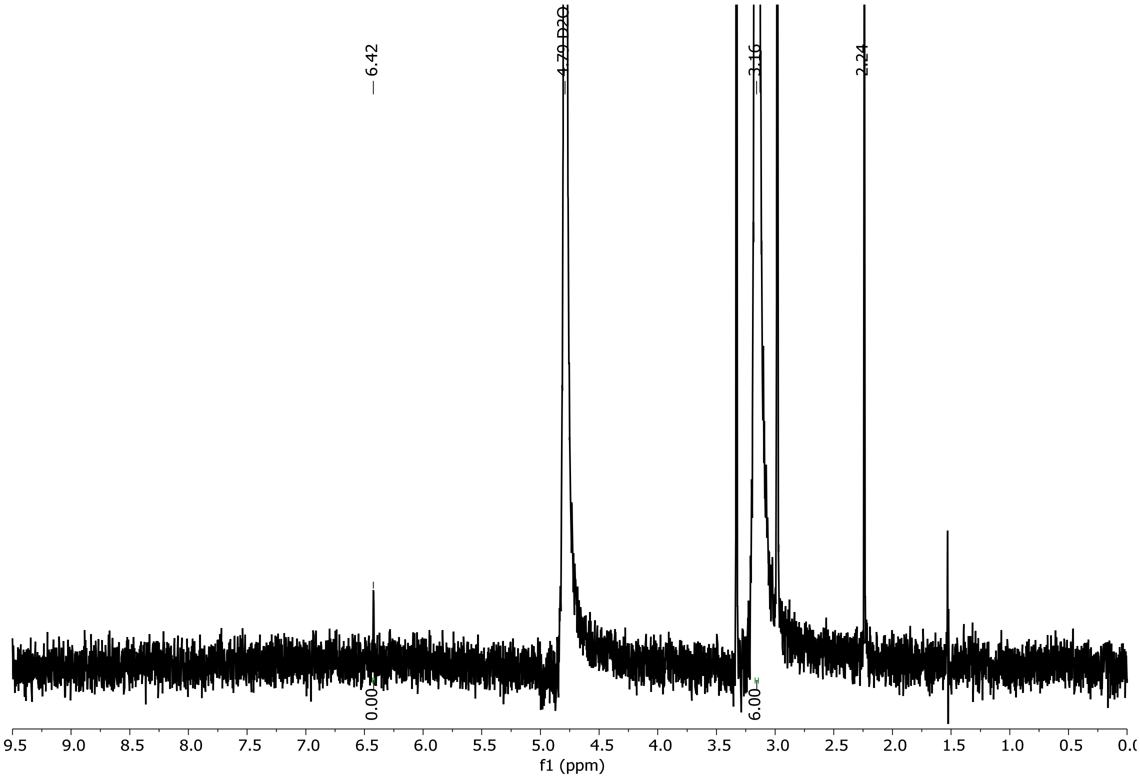

Figure S14. ^{1}H-NMR spectrum of compound **I.4d** and dimethyl sulfone.

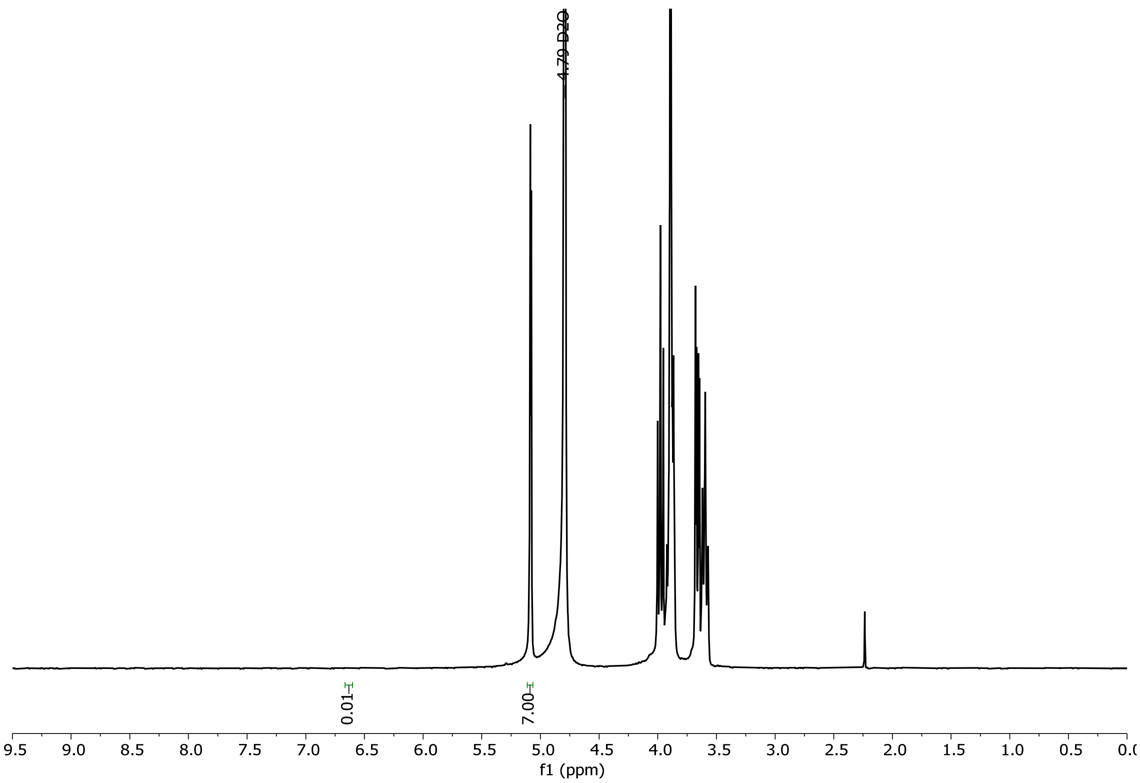

Figure S15. ^{1}H-NMR spectrum of compound **I.4d** and β-CD.

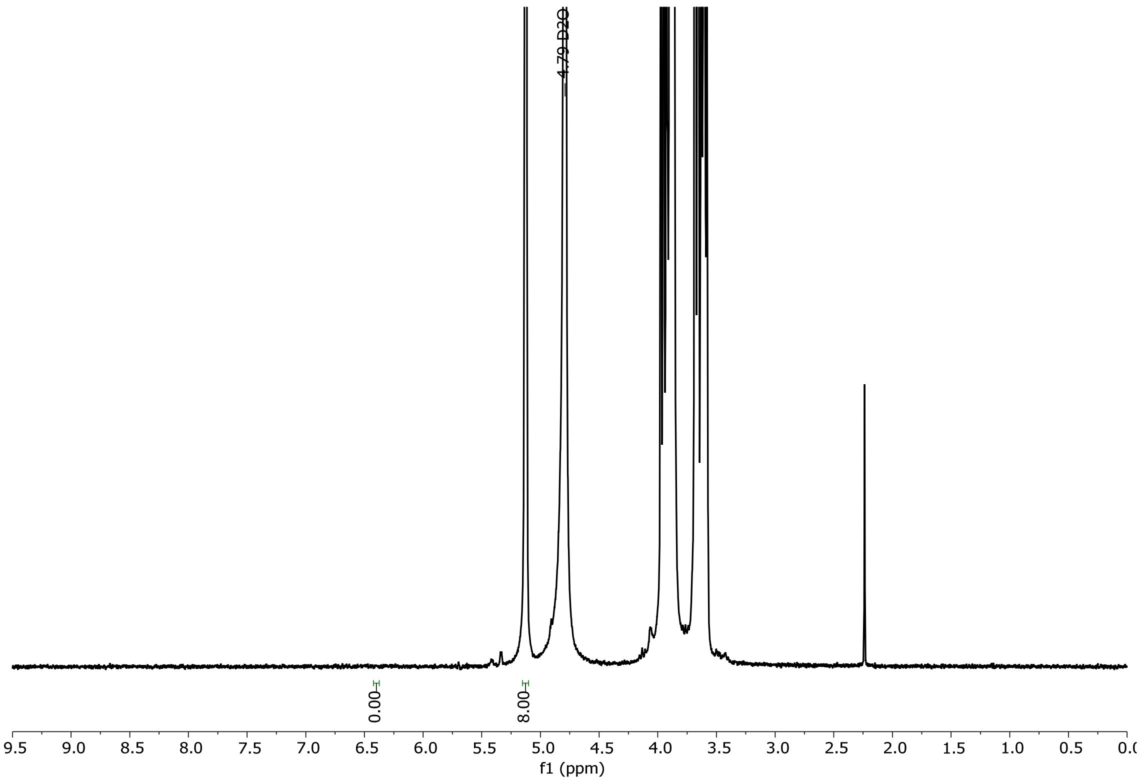

Figure S16. ^{1}H-NMR spectrum of compound **I.4d** and γ-CD.

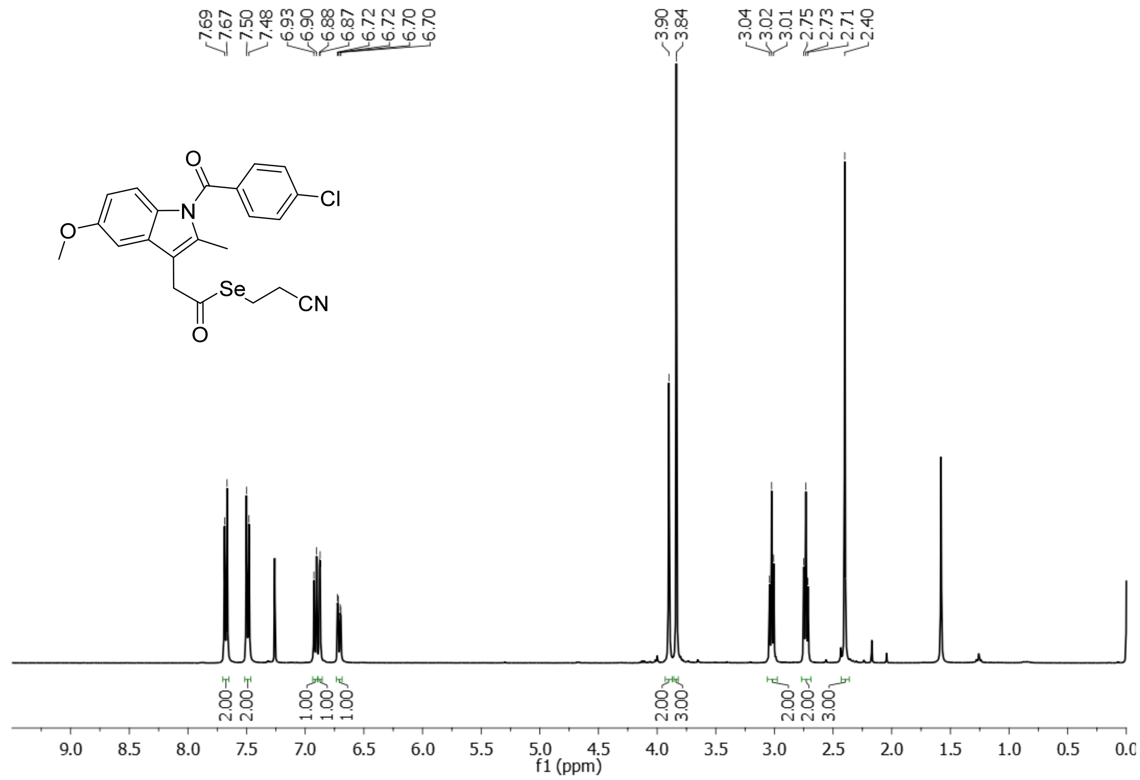

Figure S17. ¹H-NMR spectrum of compound I.**4e**.

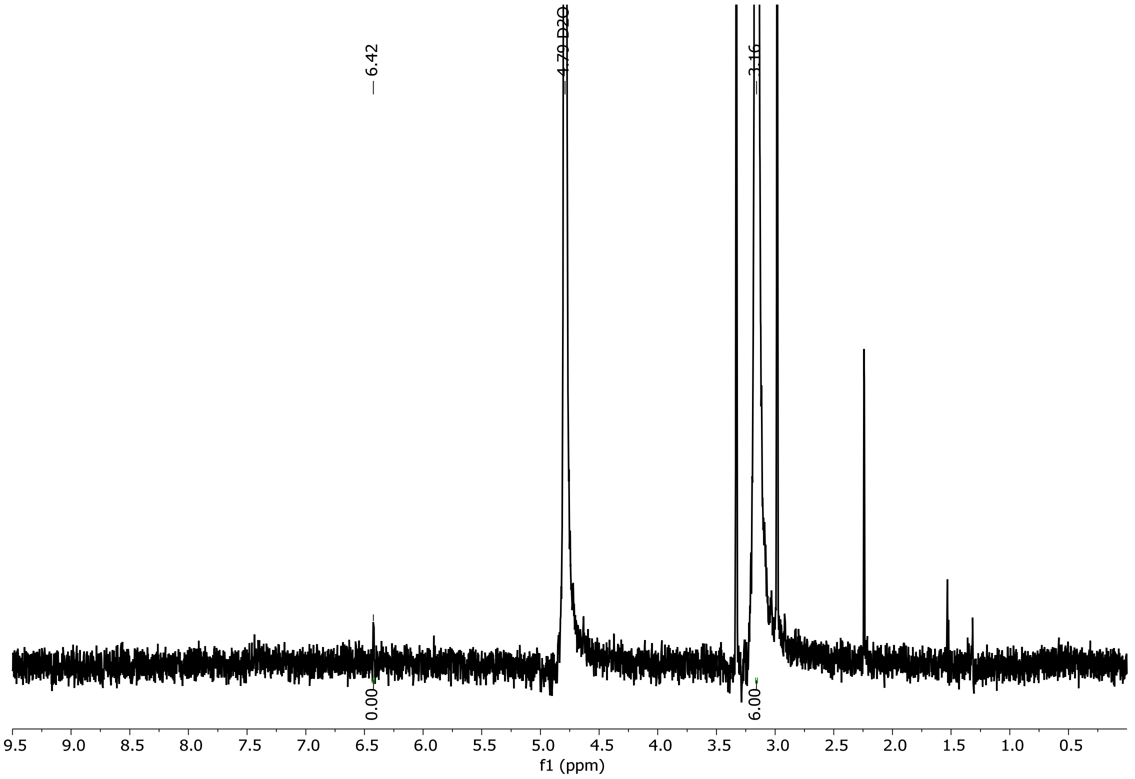

Figure S18. ¹H-NMR spectrum of compound **I.4e** and dimethyl sulfone.

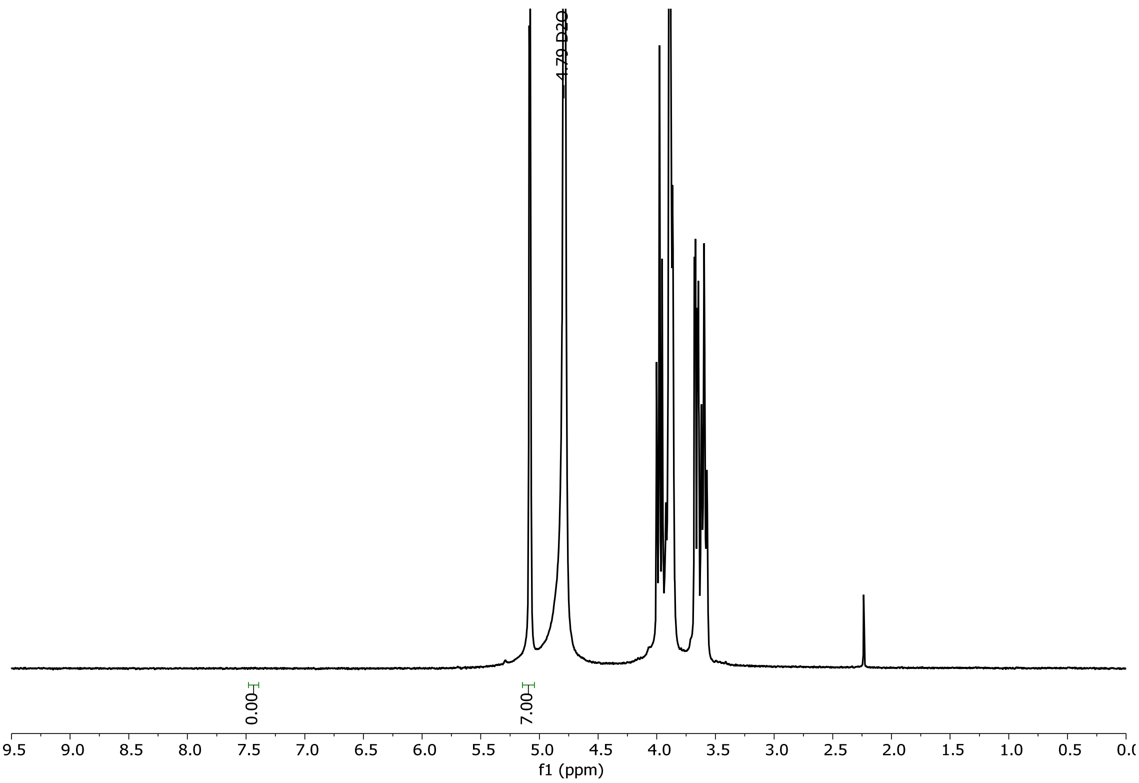

Figure S19. ^{1}H-NMR spectrum of compound **I.4e** and β-CD.

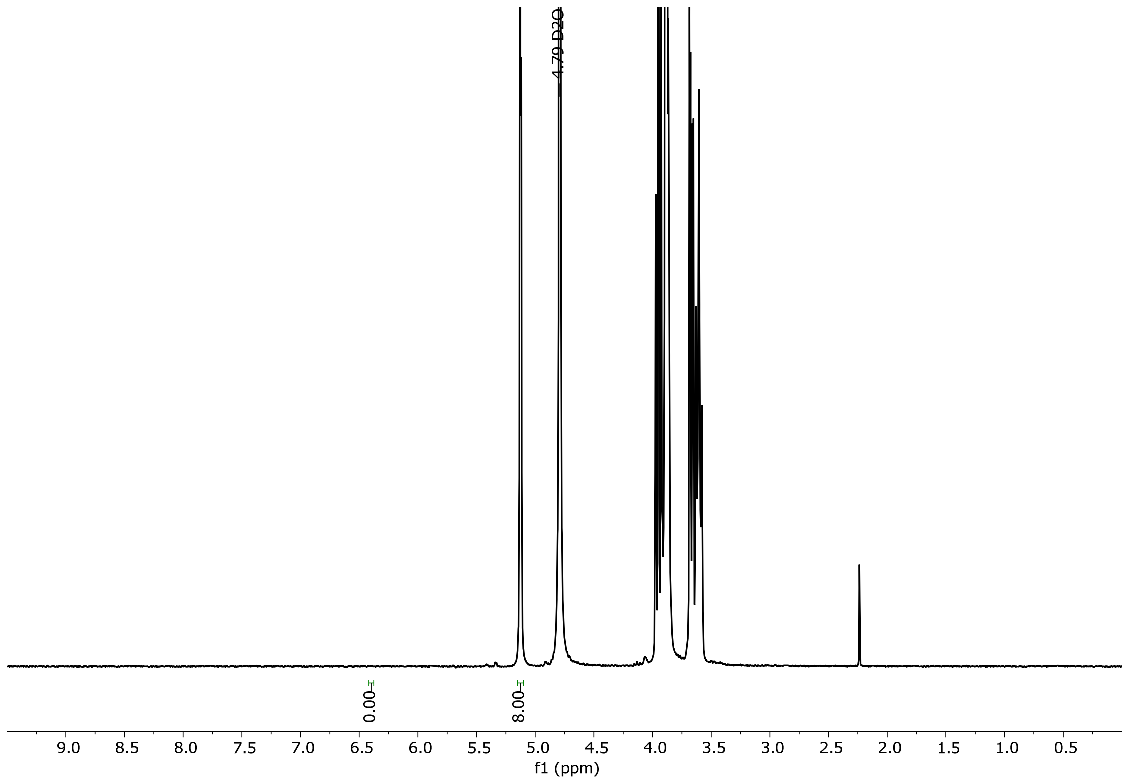

Figure S20. ^{1}H-NMR spectrum of compound **I.4e** and γ-CD.

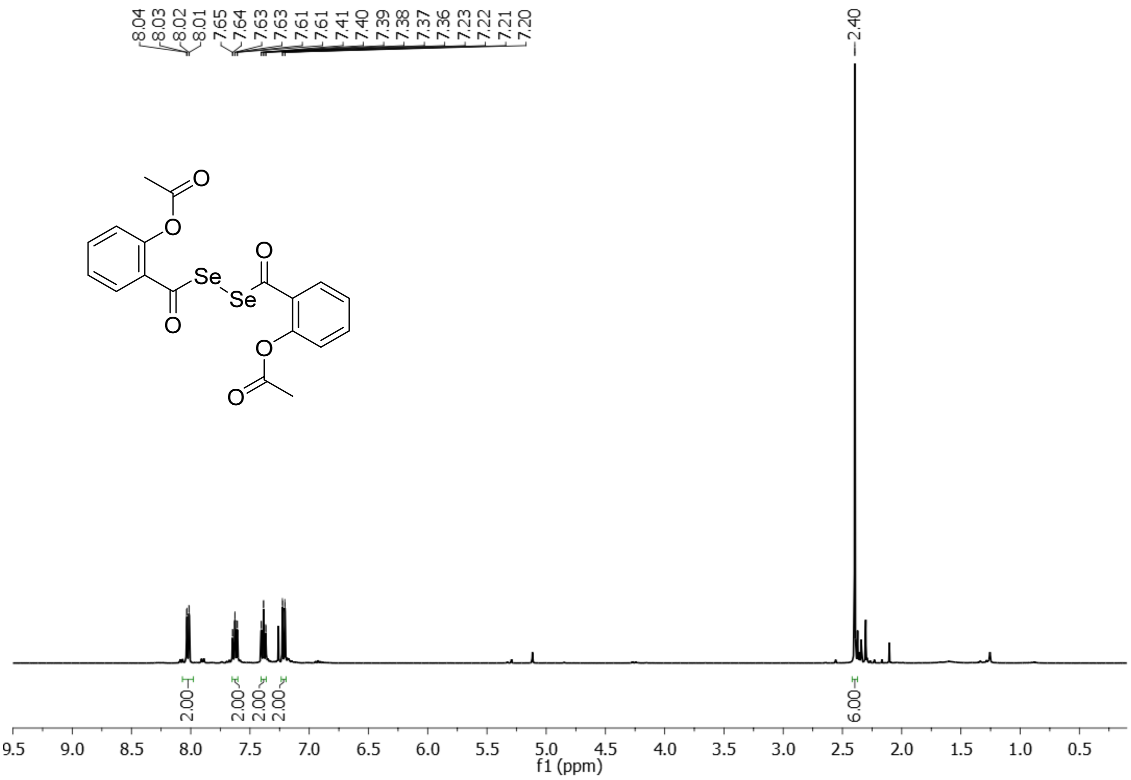

Figure S21. ^{1}H-NMR spectrum of compound **II.1**.

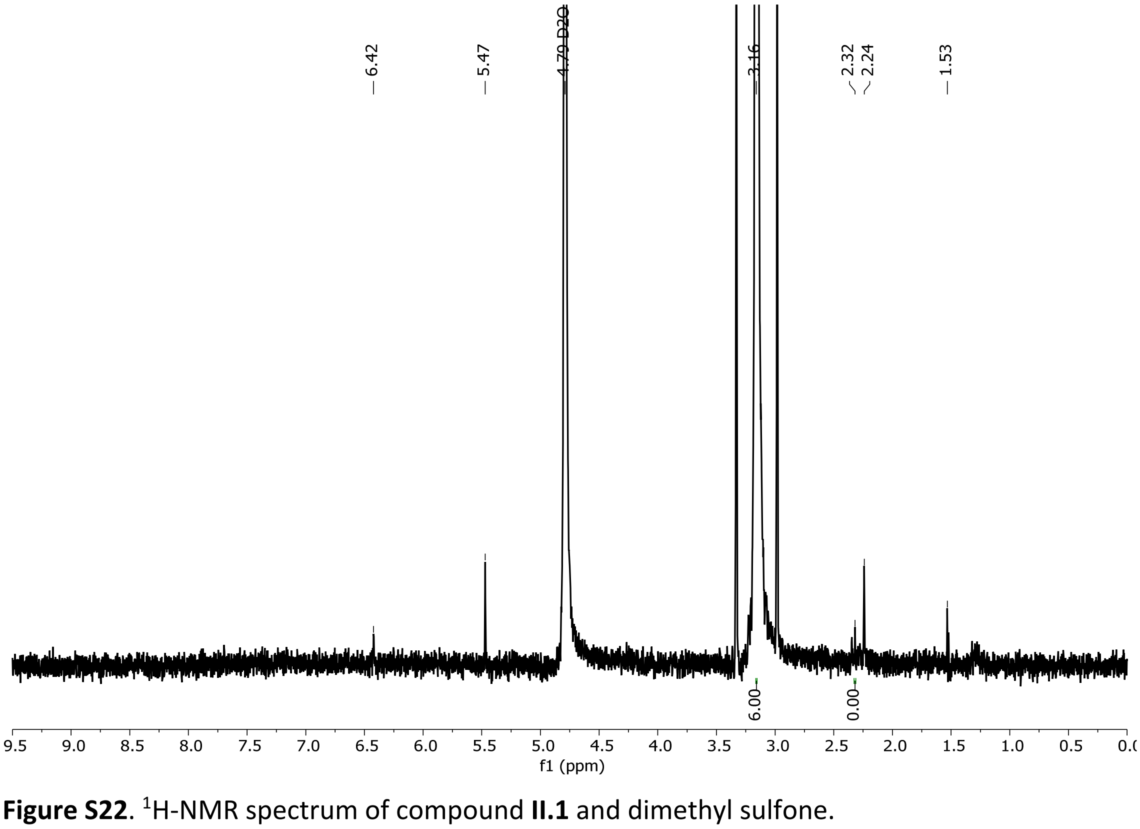

Figure S22. ^{1}H-NMR spectrum of compound **II.1** and dimethyl sulfone.

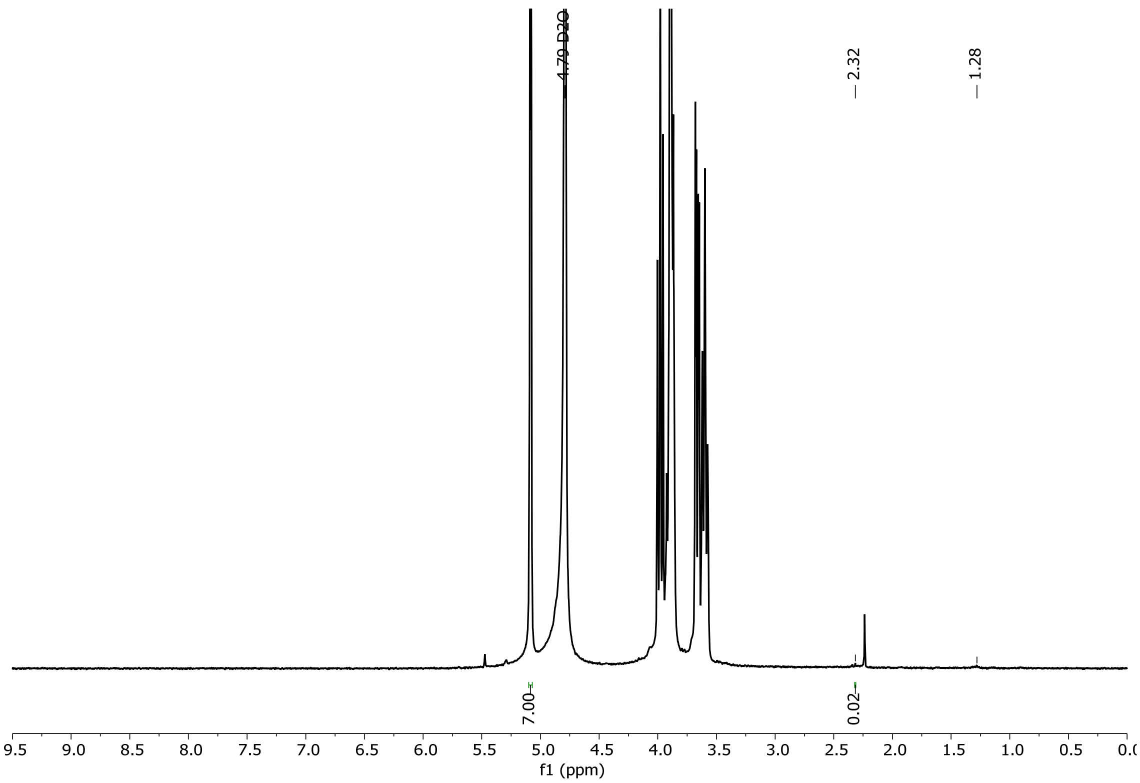

Figure S23. ^{1}H-NMR spectrum of compound **II.1** and β-CD.

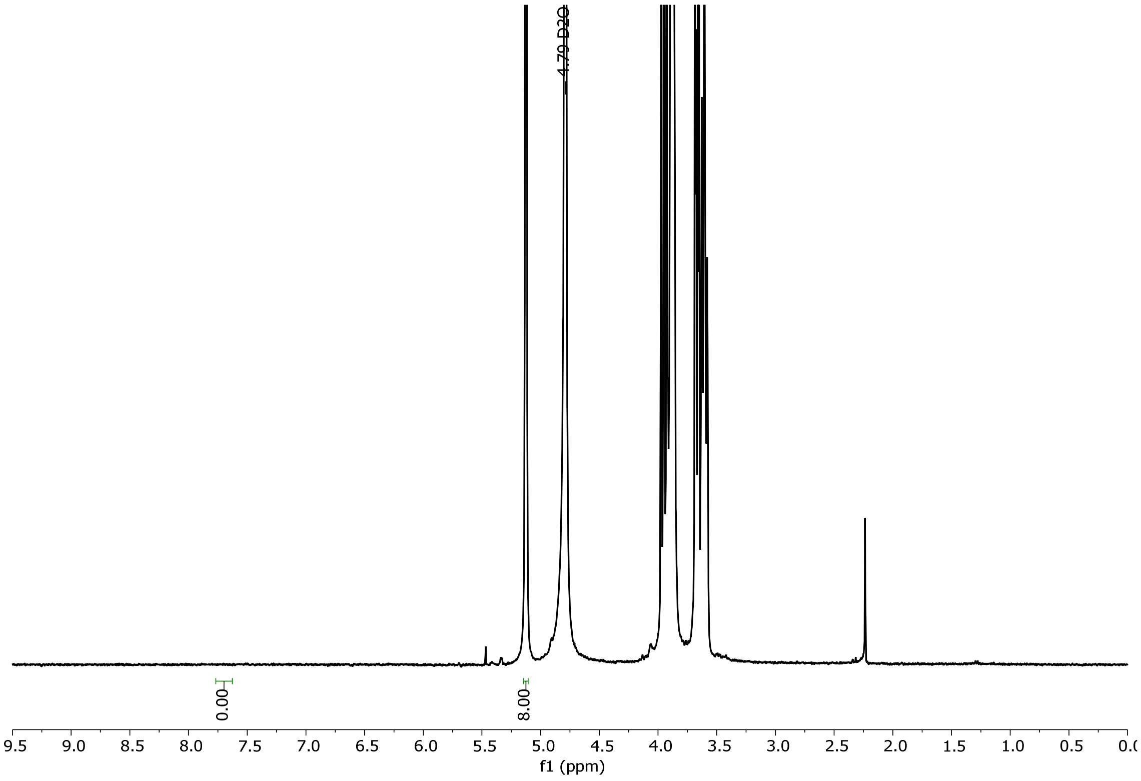

Figure S24. ^{1}H-NMR spectrum of compound **II.1** and γ-CD.

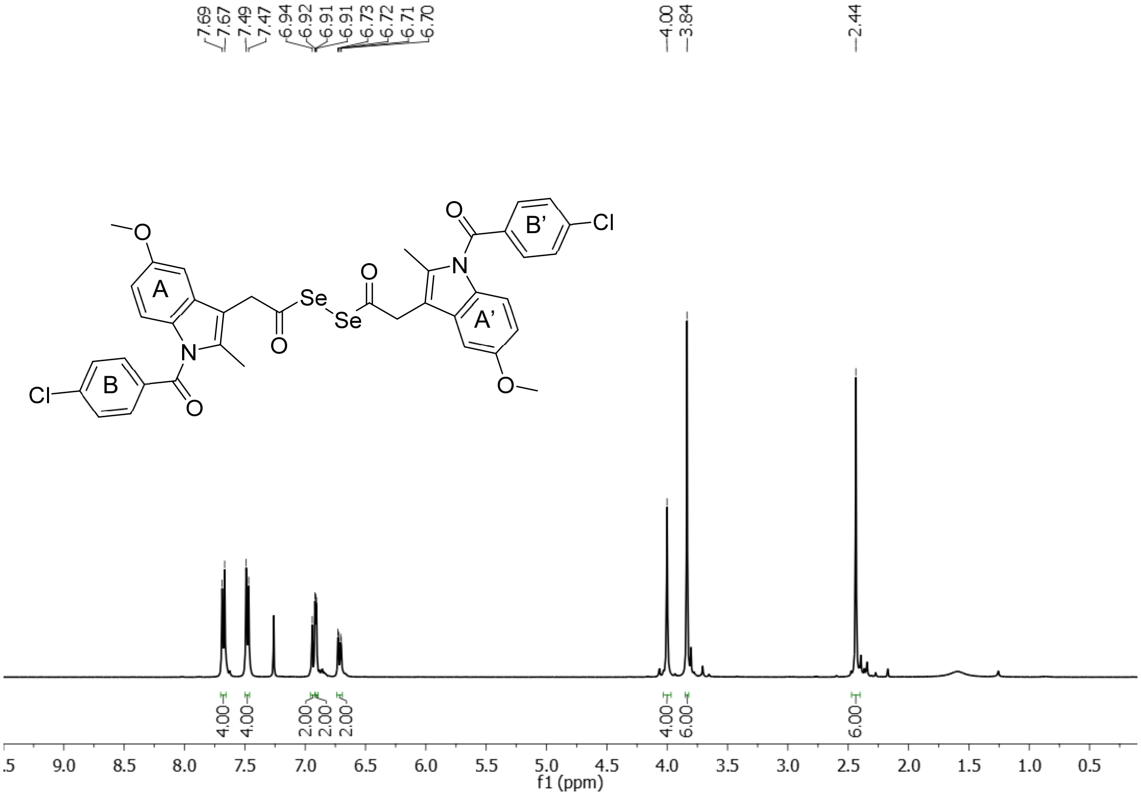

Figure S25. ^{1}H-NMR spectrum of compound **II.2**.

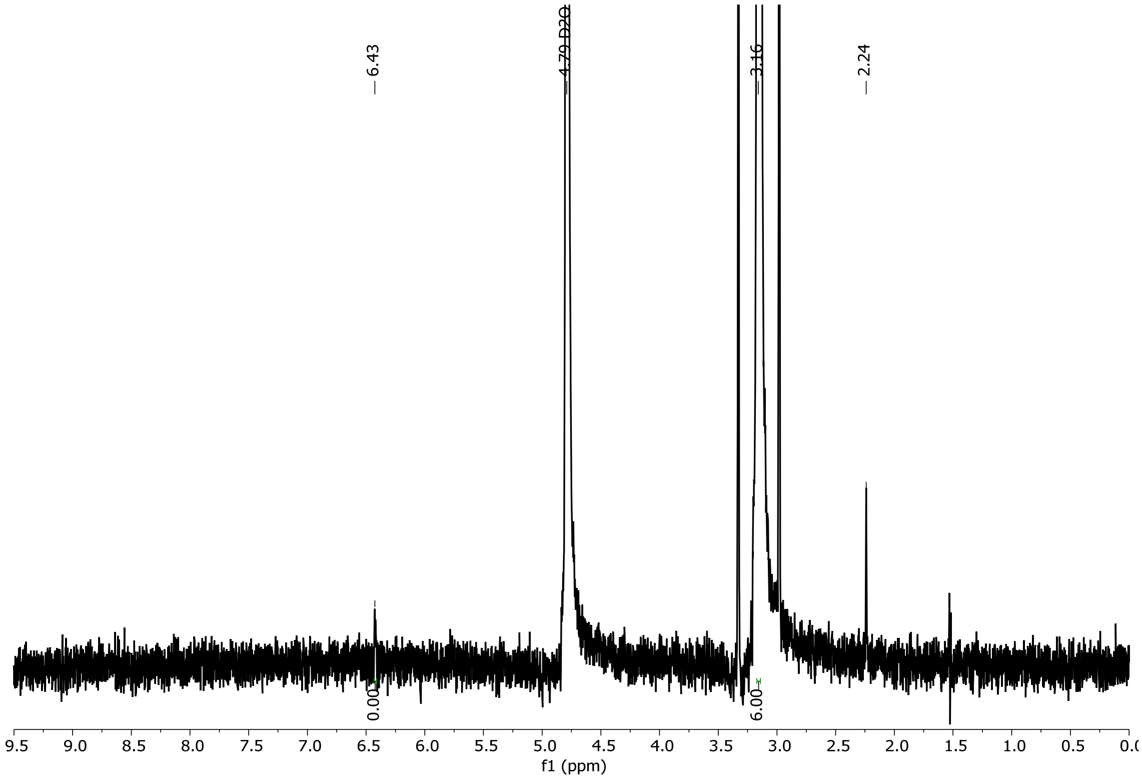

Figure S26. ^{1}H-NMR spectrum of compound **II.2** and dimethyl sulfone.

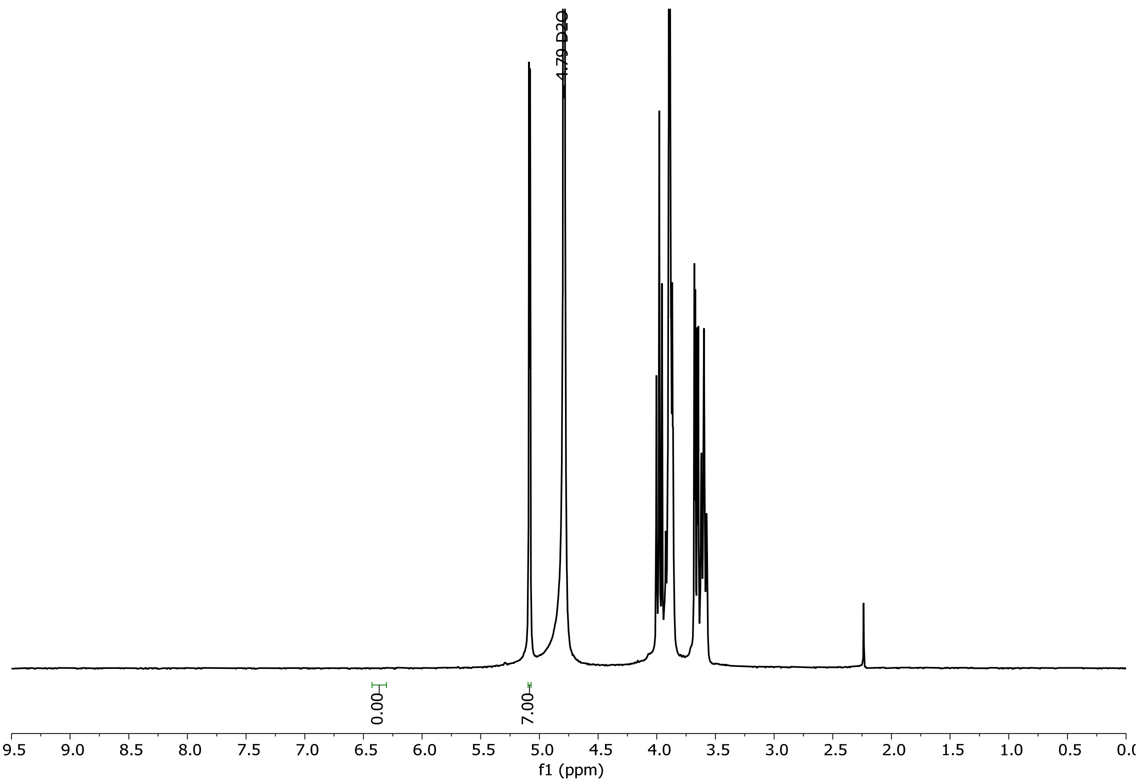

Figure S27. ^{1}H-NMR spectrum of compound **II.2** and β-CD.

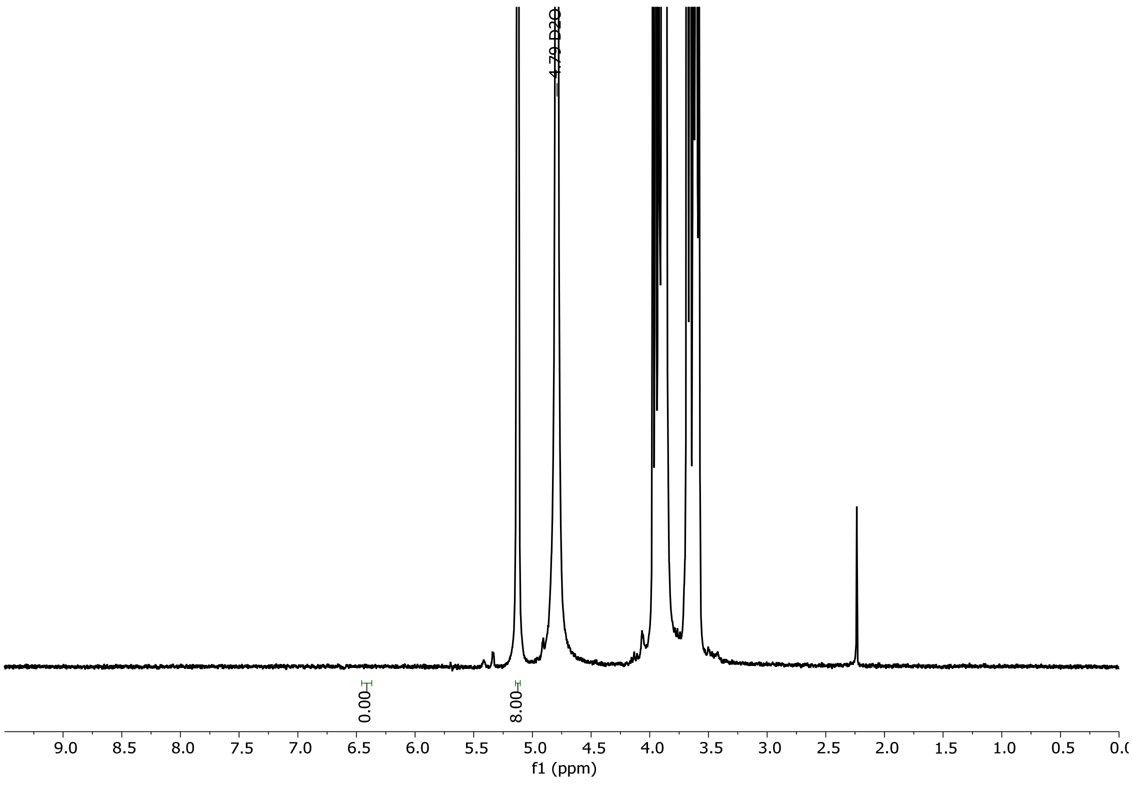

Figure S28. ^{1}H-NMR spectrum of compound **II.2** and γ-CD.

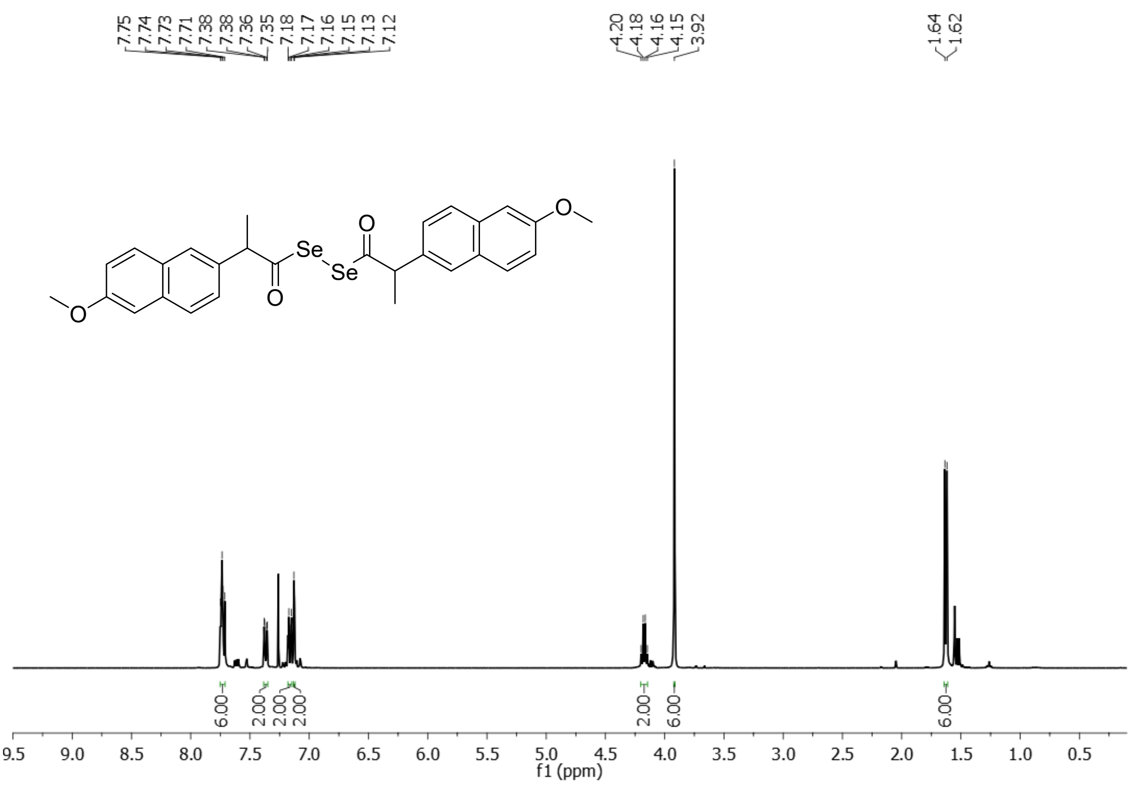

Figure S29. ^{1}H-NMR spectrum of compound **II.3**.

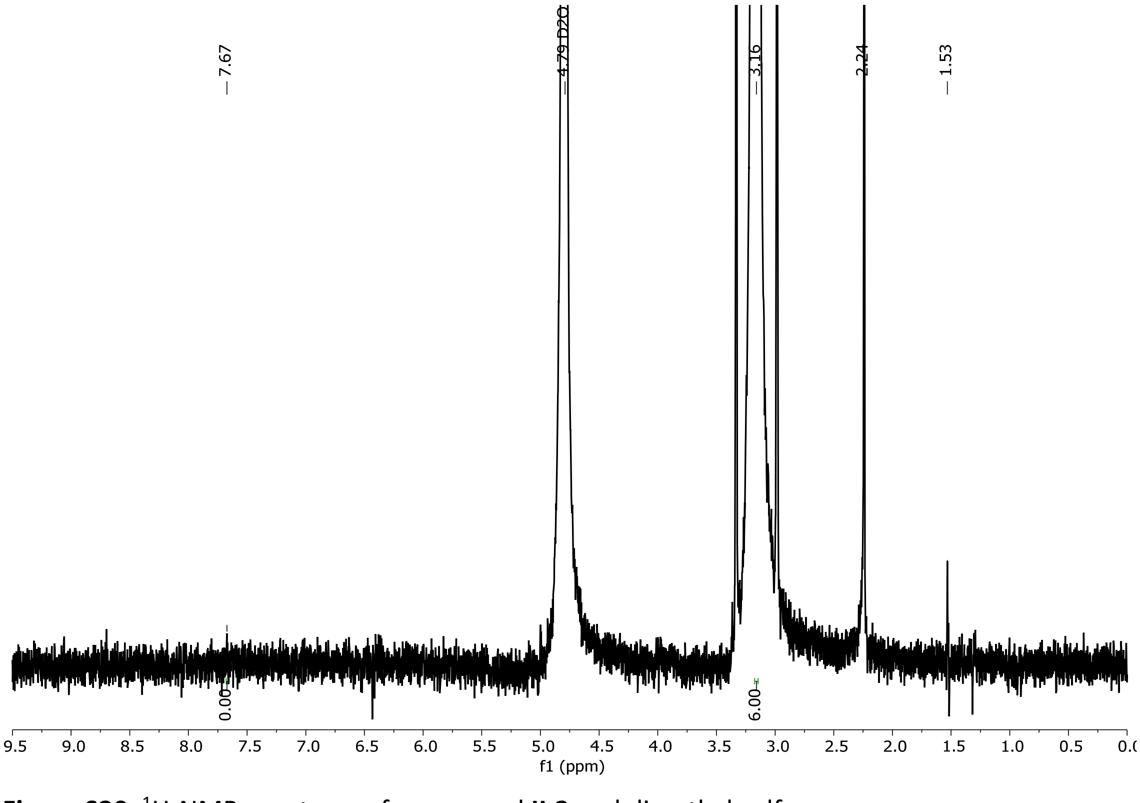

Figure S30. ^{1}H-NMR spectrum of compound **II.3** and dimethyl sulfone.

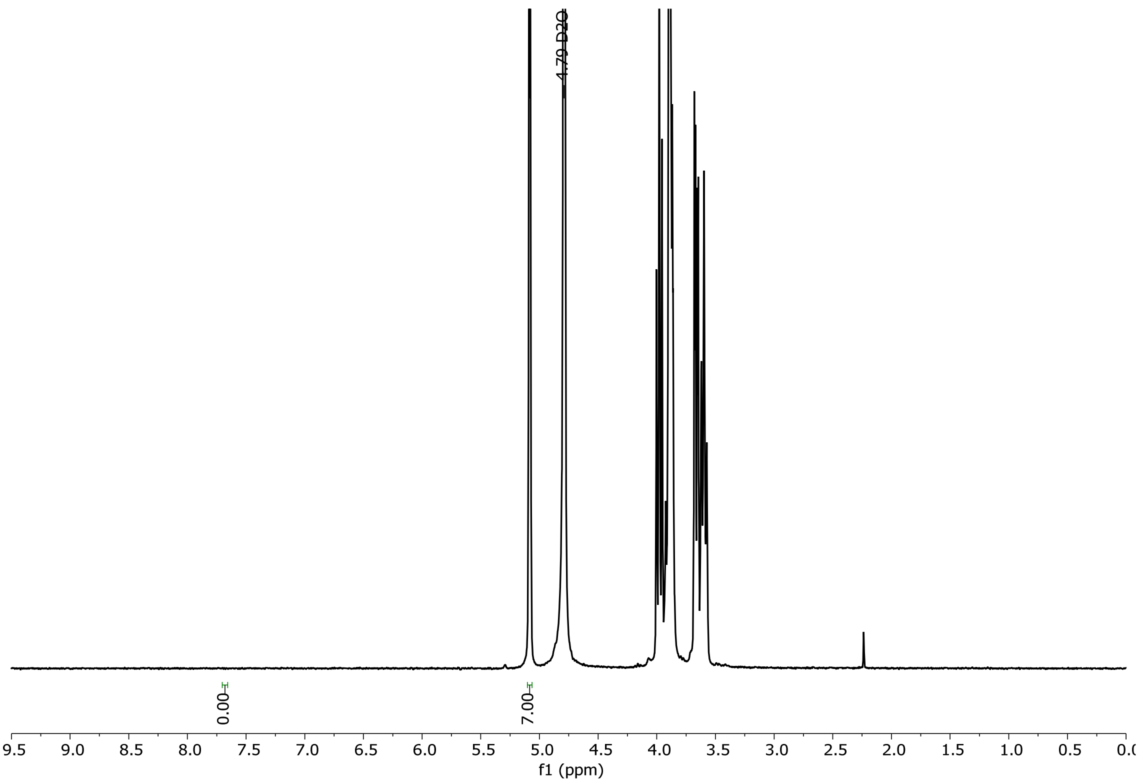

Figure S31. ^{1}H-NMR spectrum of compound **II.3** and β-CD.

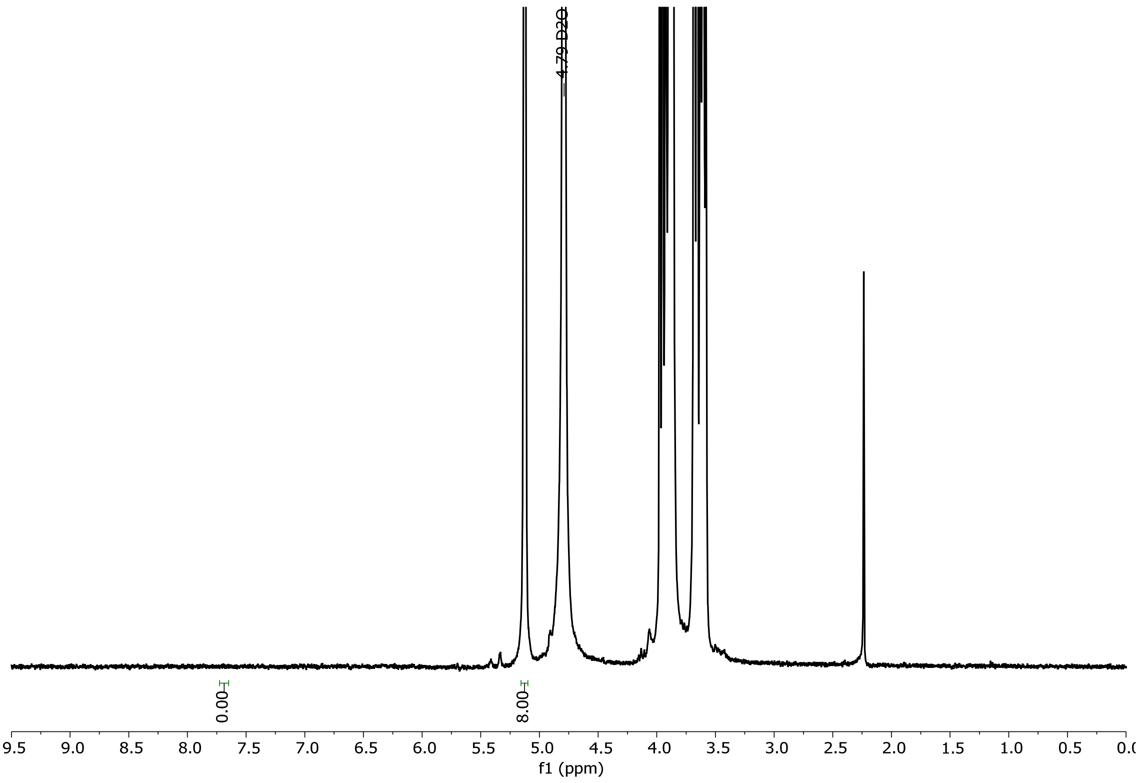

Figure S32. ^{1}H-NMR spectrum of compound **II.3** and γ-CD.

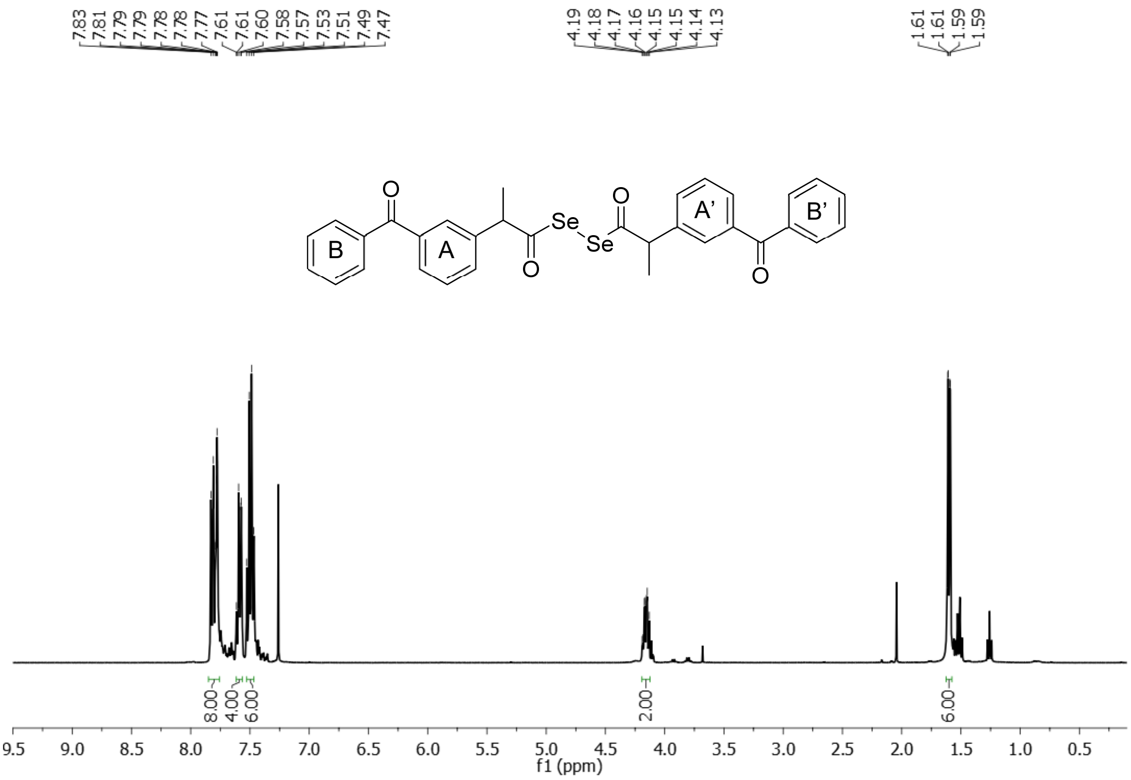

Figure S33. ¹H-NMR spectrum of compound **II.4**.

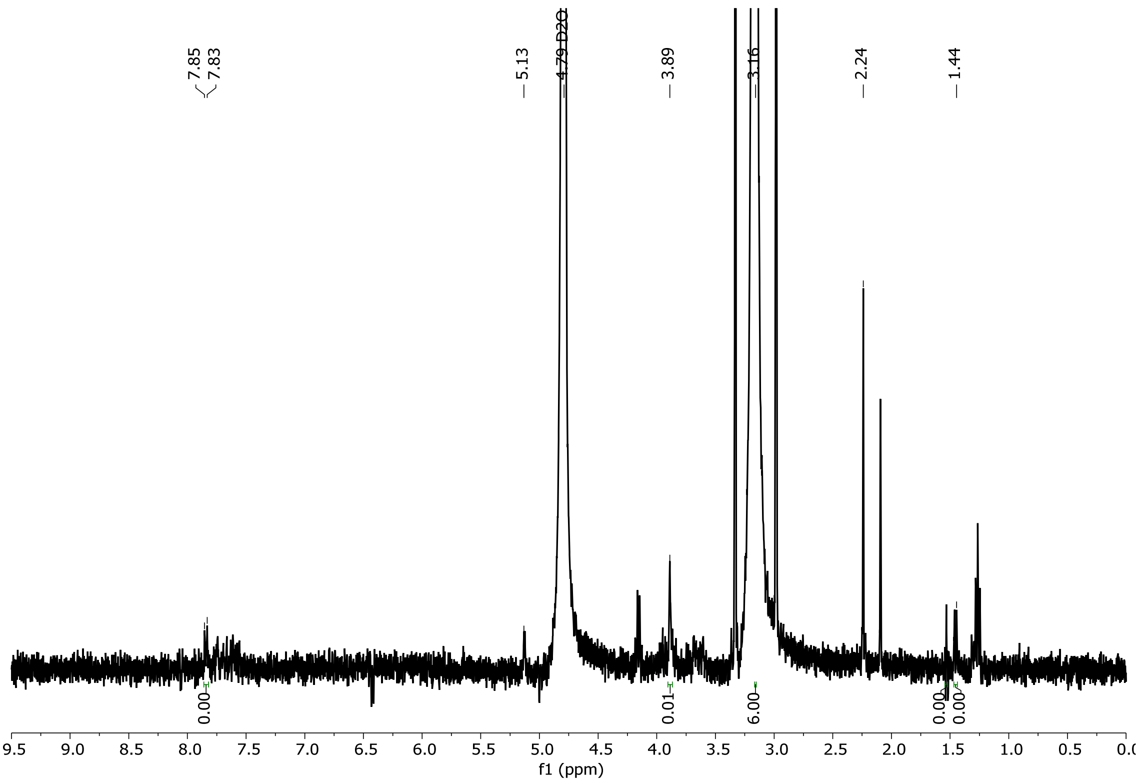

Figure S34. ¹H-NMR spectrum of compound **II.4** and dimethyl sulfone.

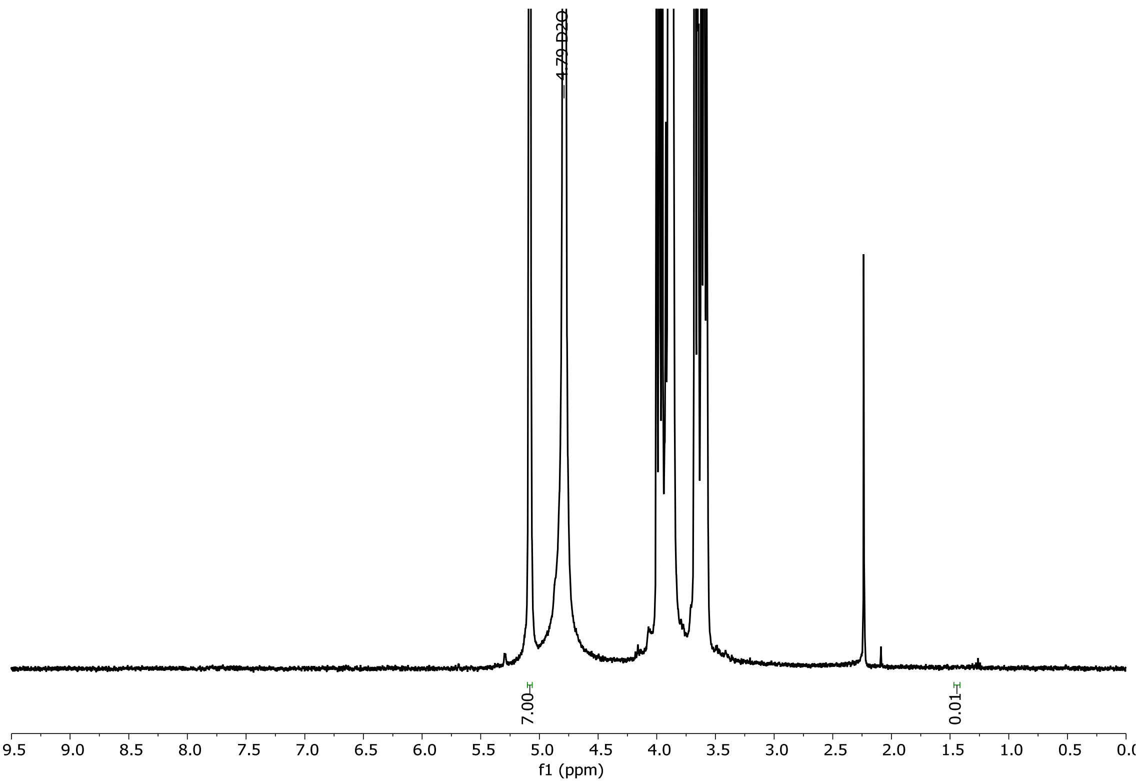

Figure S35. ^{1}H-NMR spectrum of compound **II.4** and β-CD.

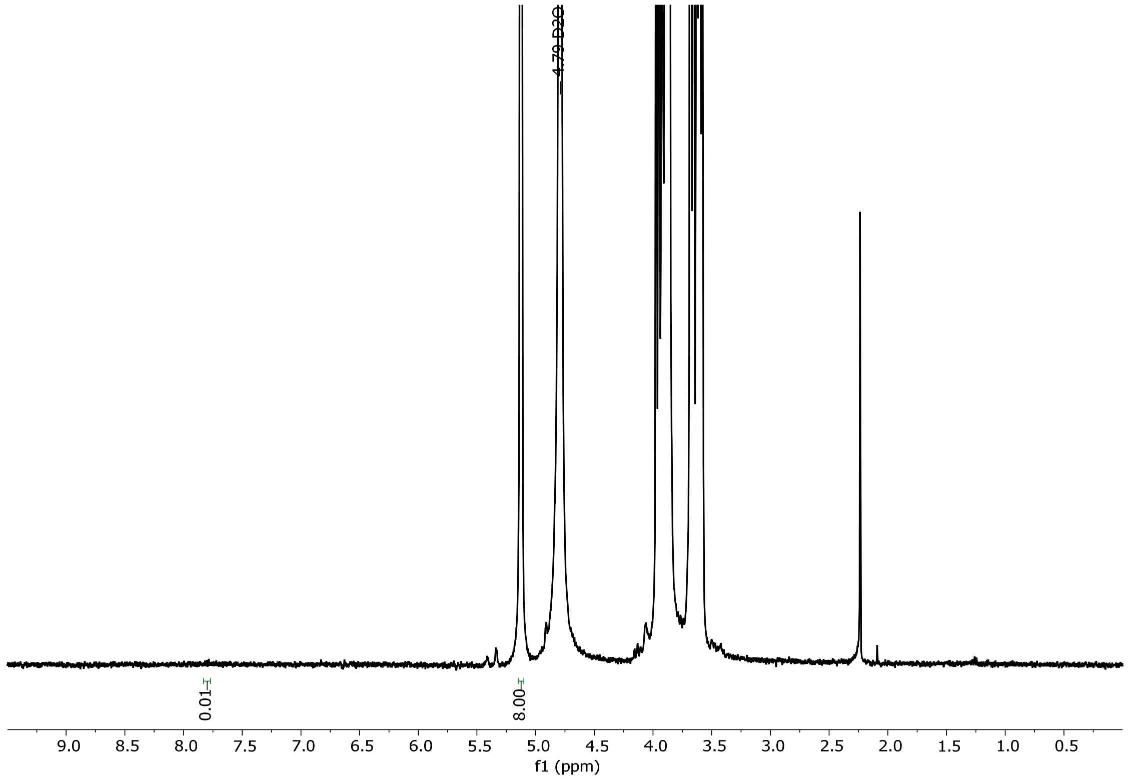

Figure S36. ^{1}H-NMR spectrum of compound **II.4** and γ-CD.

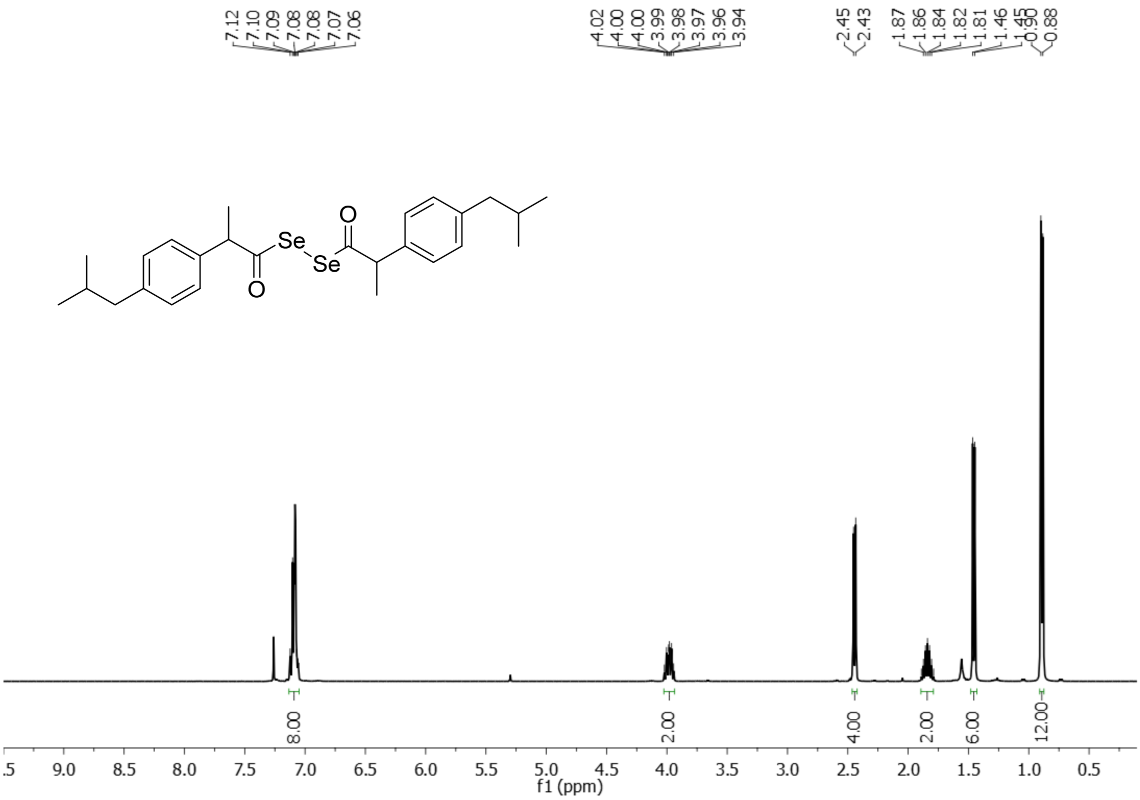

Figure S37. ^{1}H-NMR spectrum of compound **II.5**.

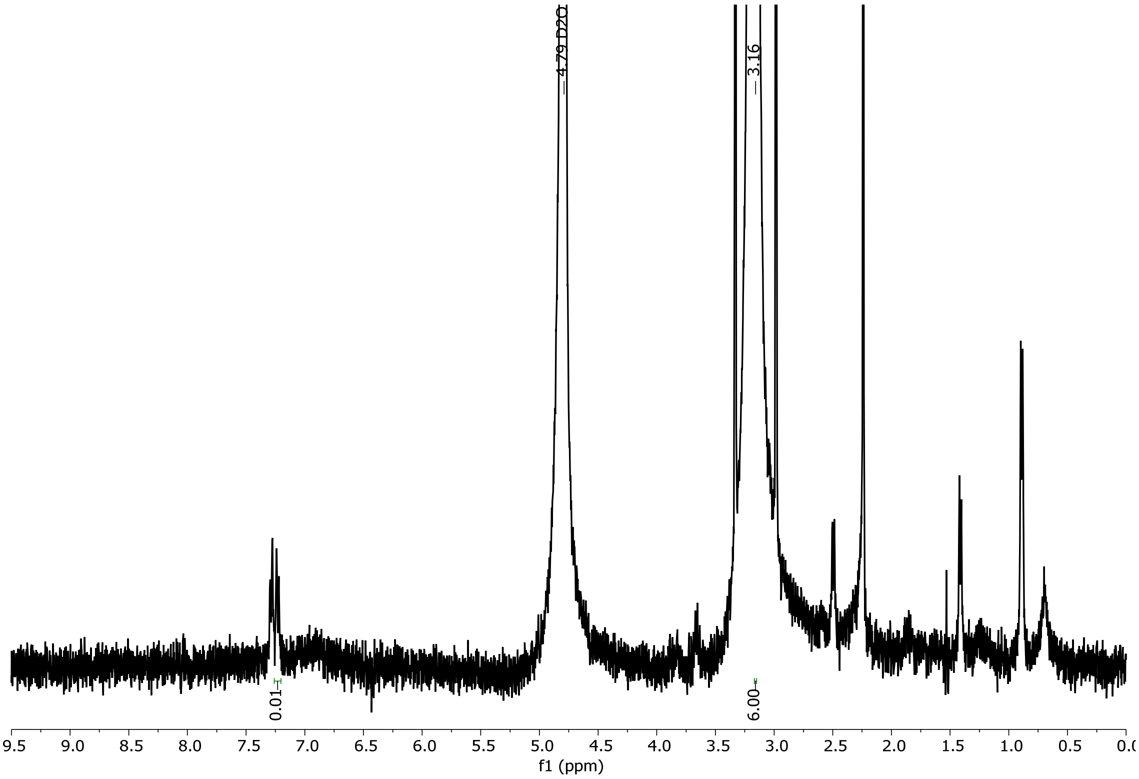

Figure S38. ^{1}H-NMR spectrum of compound **II.5** and dimethyl sulfone.

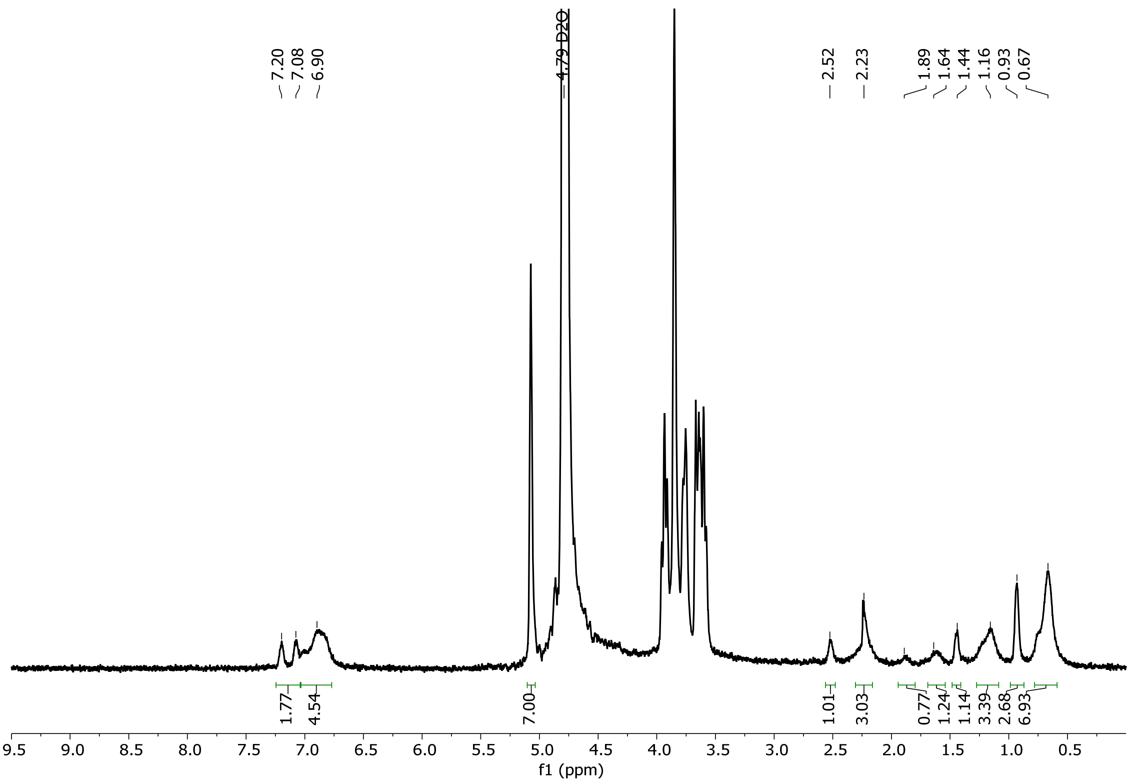

Figure S39. ^{1}H-NMR spectrum of compound **II.5** and β-CD.

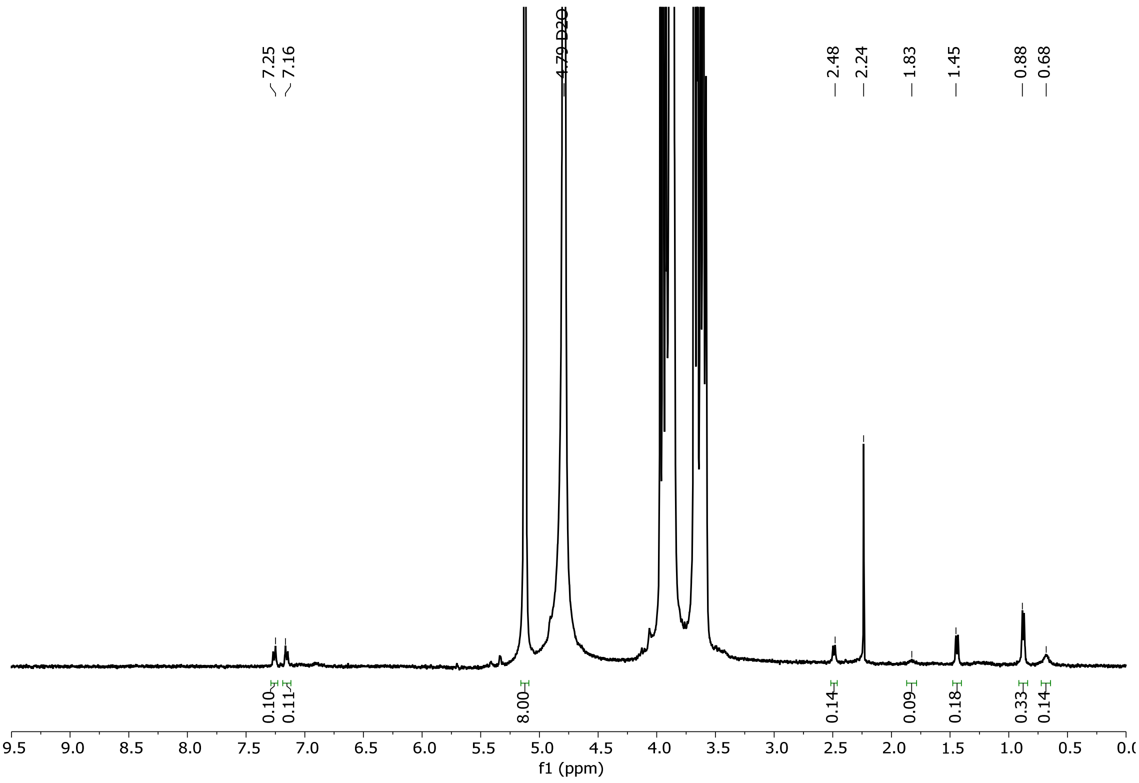

Figure S40. ^{1}H-NMR spectrum of compound **II.5** and γ-CD.

☙ Complex II.5:β-CD

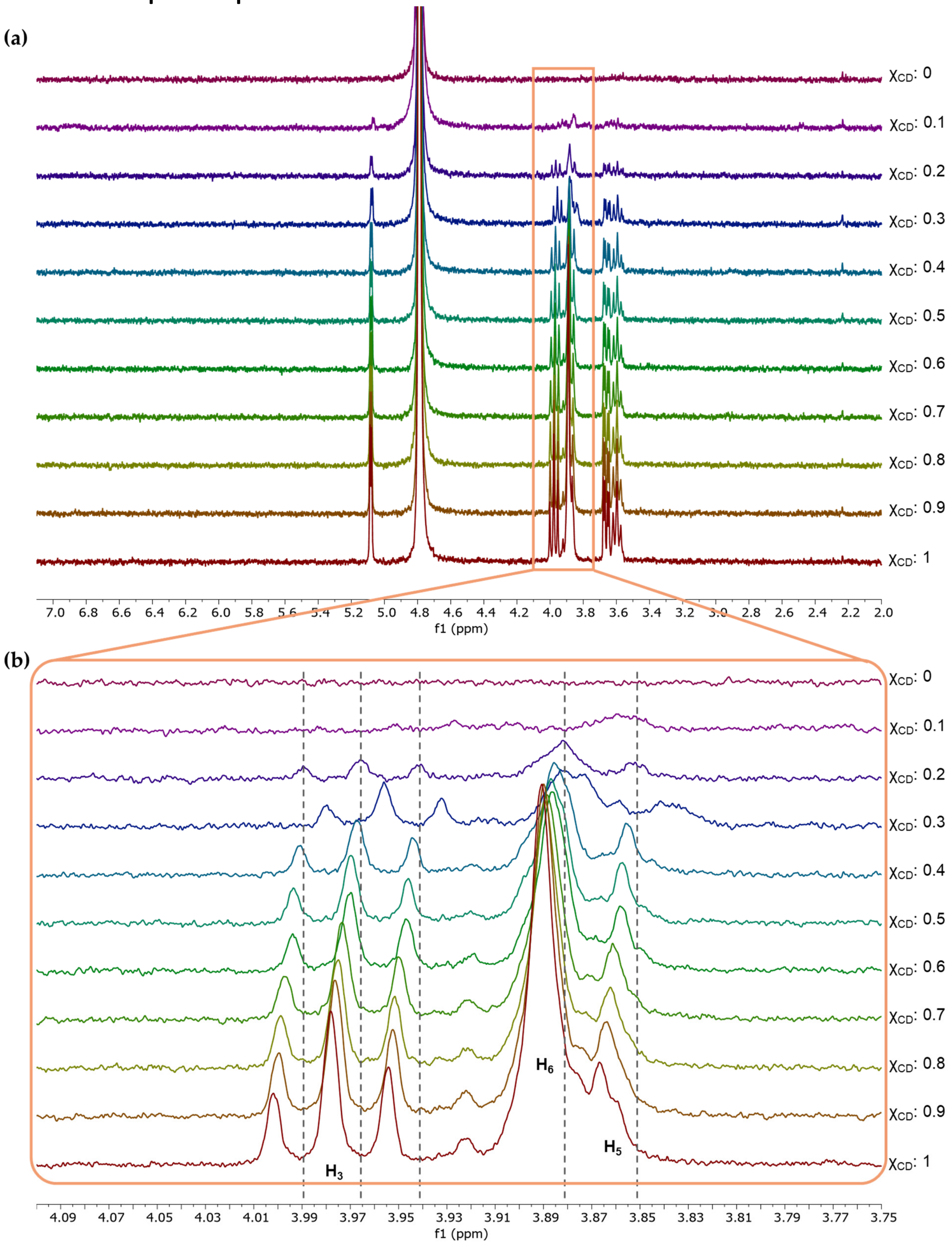

Figure S41. (**a**) ^{1}H-NMR spectra for different molar fractions of the complex **II.5**:β-CD and (**b**) expansion of the region including protons H_3, H_5 and H_6 of the β-CD.

☙ Computational data

Table S1. Scores for best poses with β-CD (London GBVI, explicit solvent).

Ref.	S	rmsd_refine	E_score1	E_refine	E_score2
**I.3e**	-5.7072	0.9549	-9.1928	-28.8446	-5.7072
**I.4a**	-6.5707	1.3715	-11.1362	-35.2667	-6.5707
**I.4b**	-6.6023	2.5629	-9.1989	-37.9295	-6.6023
**I.4d**	-6.3956	2.1327	-13.9270	-32.9405	-6.3956
**I.4e**	-6.6008	2.0304	-9.4502	-36.2130	-6.6008
**II.1**	-6.3968	2.1026	-8.5649	-30.1874	-6.3968
**II.2**	-7.5321	3.3643	-7.8035	-42.4309	-7.5321
**II.3**	-6.5140	2.3443	-7.5844	-39.5321	-6.5140
**II.4**	-6.6059	1.4075	-7.6007	-41.1813	-6.6059
**II.5**	-6.5295	2.3124	-7.6958	-37.8857	-6.5295

Table S2. Scores for best poses with γ-CD (London GBVI, explicit solvent).

Ref.	S	rmsd_refine	E_score1	E_refine	E_score2
**I.3e**	-8.0412	1.9909	-3.1866	-33.4640	-8.0412
**I.4a**	-9.6127	2.2584	-1.8757	-34.6887	-9.6127
**I.4b**	-8.8817	1.9890	-1.9153	-32.3470	-8.8817
**I.4d**	-8.8520	4.6771	-2.2712	-35.2892	-8.8520
**I.4e**	-9.1430	4.9243	-2.1714	-36.6787	-9.1430
**II.1**	-8.1714	1.2808	-3.8911	-35.5261	-8.1714
**II.2**	-10.1487	1.6912	1.8735	-42.3182	-10.1487
**II.3**	-8.9557	2.0750	0.2874	-38.5811	-8.9557
**II.4**	-9.1211	2.6151	-0.5199	-42.0503	-9.1211
**II.5**	-7.9440	2.0826	2.5007	-34.7207	-7.9440

Table S3. Energy values for best poses of selected compounds (London GBVI, explicit solvent).

Ref. complex	E all
**β-CD**	
**I3e_best_solvent**	-257.77
**II5_01_best_solvent**	-465.87
**II5_02_best_solvent**	-463.43
**γ-CD**	
**I3e_best_solvent**	-293.76
**II5_01_best_solvent**	-508.33
**II5_02_best_solvent**	-507.18
